# Self-reported cheating among medical students: An alarming finding in a cross-sectional study from Saudi Arabia

**DOI:** 10.1371/journal.pone.0194963

**Published:** 2018-03-29

**Authors:** Hamza Mohammad Abdulghani, Shafiul Haque, Yousef Abdullah Almusalam, Saleh Lafi Alanezi, Yazeed Abdulaziz Alsulaiman, Mohammad Irshad, Shaffi Ahmed Shaik, Nehal Khamis

**Affiliations:** 1 Department of Medical Education, College of Medicine, King Saud University, Riyadh, Saudi Arabia; 2 Research and Scientific Studies Unit, College of Nursing and Allied Health Sciences, Jazan University, Jazan, Saudi Arabia; 3 Department of Family & Community Medicine, College of Medicine, King Saud University, Riyadh, Saudi Arabia; Kyoto University, JAPAN

## Abstract

Academic misconduct/dishonesty has become widespread behavior among many university students across the globe, and medical education is not an exception. Until recently, few efforts have been made to study the dishonest behavior in Middle-Eastern universities. This study examined the prevalence and predisposing factors of cheating among medical students in Saudi Arabia and suggests suitable preventive measures. A cross-sectional survey-based study was conducted at a government medical college during the 2014–2015 academic year. The response rate was 58.5% (421/720). The overall cheating behavior practiced by the participants was 29%, predominantly by male students. High GPA scoring students were the least likely to cheat. The participants living with their families were more likely to cheat compared to those who were living apart from their families. The reasons participants gave to justify their cheating behavior included getting better grades, passing the course, and lacking preparation while still recognizing that cheating is a ‘mistake.’ Overall, significant academic misconduct concerning cheating was found among the Saudi medical students; this misconduct is alarming in a reputable government institution. The implementation of strict punishments, requiring ethical courses and creating ethical awareness by exploiting the potential of Islamic religious belief might help to control this problem.

## Introduction

Academic fraud, academic misconduct, or academic dishonesty. All these terms hold similar meanings and have become widespread behavior, especially in the form of plagiarism, lying, and cheating, among university students in almost all subject areas, and medical science education is not an exception, regardless of the implementation of various preventative strategies [1,2,3]. Such improper behavior severely affects the knowledge of future doctors and therefore the quality of healthcare [4].

Earlier studies reported a high prevalence of dishonest behavior in medical schools globally [5,6,7]. Fraudulent or dishonest academic conduct was found to be prevalent among half (approximately 56%) of the medical students according to a Canadian study [8]. A study from the United Kingdom reported that dishonest conduct is prevalent among pharmacy students, and male students are more likely to confess to academic dishonesty as opposed to their female counterparts [9]. Cheating, which is a major form of academic misconduct, was also prevalent among students at Harvard University [3]. A North American study by McCabe (2005) reported that the prevalence of cheating and dishonesty was approximately 33% [1]. Students who passed an exam confessed that they received some pertinent information about the exam in advance. The study by McCabe (2005) also reported that most of the cheating was on written assignments [1]. In an Iranian study, Mortaz Hejri et al. (2013) reported that cheating and helping others to cheat are higher during internships in the final training year compared to clerkships in the 4^th^ and 5^th^ years [10]. A study from Croatia reported that cheating is prevalent among medical students, and signing attendance on behalf of other students is the most frequent kind of academic misconduct. Additionally, it is the only type of cheating, and most of the students agreed and considered it a normal behavior [5]. The persistence of cheating behavior among students has been found to be the same both before (in previous classes, e.g., pre-university and high school) and after enrollment at the university (i.e., in medical college) [11]. Earlier studies have also found that male students are significantly more involved in academic misconduct than female students [12,13,14]. In contrast, Rennie and Rudland (2003) reported no difference between the attitude and behavior of male and female students toward academic misconduct [15]. Unfortunately, what is alarming about this cheating behavior is that it is acceptable among medical students [7,11].

A decade ago, Hrabak et al. (2004) identified various social and cultural factors that contribute to academic misconduct among different communities of students [5]. In two different studies, a heavy academic load was found to be the most common factor contributing to cheating [3,16]. Other factors identified for cheating and related to individual students are laziness, poor grades, pressure to get the work done, and irresponsibility [3]. These factors usually lead to unwanted consequences that affect the appropriate learning methods [3]. A study conducted at a dental school revealed that a high percentage of students confessed to cheating because of the academic pressure [4]. A study that reviewed the data pertaining to cheating in academic institutions over the course of a decade reported that peer pressure has a significant connection to cheating [17]. Additionally, advanced technologies used in medical education have been found to contribute to cheating behavior among medical students [18].

Previously, published studies from different areas of the world identified various forms of cheating and misconduct prevalent among university students [1,4,12,13,19,20]. However, for some students, the act of cheating is copying answers from their colleagues’ answer sheet during an examination. Forging a faculty member’s signature was found to be the most common cheating behavior in Jordan and India [5,19,20] while copying during an examination the most common way of cheating in medical school in the USA [3].

Students have different motivations to cheat. A study showed that students cheat to get better grades, to appear academically proficient or intelligent in front of their family members and friends, or to pass the course [20]. Some students justified their cheating with attitudinal and situational factors [4]. However, the overwhelming majority of the students never justified cheating [19].

It is clear that requiring a ‘course on ethics’ during medical school is not an effective means to prevent cheating [4]. The majority of students who observed cheating behavior did not report it to their faculty members, possibly because of the friendship that they had with the cheater(s) or because of the fear that their identities would be revealed [4,21].

To control the prevalence of cheating, various strategies have been suggested and implemented across the globe. Additionally, numerous studies have investigated the possible punishment options that can minimize cheating. Warnings and student counseling of the most preferred and accessible methods of controlling the prevalence of cheating for the teachers [20], but until now no acceptable progress has been reported. Therefore, the faculty members prefer more severe penalties for students involved in cheating [17,22]. The faculty members are aware of the prevalence of different types of cheating strategies, but they fail to confront it due to lack of evidence [1]. Remarkably, students’ awareness of academic integrity policies and signing an ‘Honor Codes’ document is believed to lower the occurrence of cheating among medical students [4,23].

There is a lack of research on academic misconduct (especially related to cheating behavior) in medical schools, and only scant reports are available from the universities of Middle Eastern countries. Additionally, there are currently no local or regional studies available that discuss the sensitive issue of cheating among medical students. Thus, no strict rules or punishment options have been suggested or implemented as a preventive measure for cheating. The socio-cultural background, religious belief, ethical values and attitude of the people living in the Middle East (especially Saudi Arabia) are different than that of people living elsewhere. Therefore, there is no guarantee that similar rules or punishments will work in this region. Considering the facts mentioned above about the various types of academic misconduct, the present study was conducted at a government medical college in Saudi Arabia to determine the prevalence and predisposing factors responsible for cheating among students and suggests ways to prevent this academic misconduct.

## Methods

### Context, participants, study design, and data collection

A cross-sectional, self-administered questionnaire-based study was conducted at a governmental medical college in Saudi Arabia during the 2014–2015 academic session (Supporting Information: S1 Appendix). The study population included medical students (age group 22–26) of the fourth, fifth, and final academic and internship years. To collect the data, a validated scenario-based questionnaire was adapted from a previously published study [6]. The questionnaire had three sections: student’s demographic information, student’s behavior, and student’s attitude. The first section consisted of questions related to personal data and social background, including the participants’ academic achievement levels in the form of their grade point average (GPA). The second section was comprised of multiple scenarios in the English language, and for each scenario, there were two questions to answer (1. Is the student wrong? Yes/No; 2. Have you done or considered doing the same? Yes/No). All the scenarios were related to academic misconduct in the form of plagiarism, lying, and cheating. The scenarios tested whether the student agrees or disagrees with the behavior, or if he/she would consider doing the same in the future. Likewise, in the third section, multiple choice questions were posed for the students to justify their behavior(s) if they confessed to cheating during their academic years. Some blank spaces were provided to add other justifications or suggestions for the prevention of such problematic behavior(s). The survey questionnaire was distributed to the medical students and interns by sending messages to their emails and mobile phones via WhatsApp (social media). The email and WhatsApp messages were specific to each student and intern and were sent separately. Two reminders after one week and two weeks were sent to the non-responsive participants following the non-compliance.

### Statistical analysis

The survey questionnaire was collected and reviewed, and the data were extrapolated and tabulated for the analysis. All the statistical analyses involved in the present study were performed using the SPSS Version 21 software program [24]. Descriptive statistics were employed to measure the percentages/frequencies, and a regression analysis test was used to evaluate the association between different variables under consideration. During the analysis, the statistical significance level was p-value <0.05.

### Ethical approval

This study was approved by the Ethical Review Committee of the participating university medical college in Saudi Arabia. Written consent was provided to all the participants, and the participation in the study was voluntary. The purpose of the study was explained to each participant, and the confidentiality and anonymity of each participant were maintained by assigning a code number.

## Results

The total number of respondents in our study was 421 out of 720, with a response rate of 58.5%. Of 421 respondents, 129 (30.6%) participants were from the fourth year, 152 (36%) were from the fifth year, and 140 (33.2%) were from the internship year.

The summary of the personal data and the social background of the participants is given in Table 1. The majority of the participants were from an urban background (386/421; 91.7%), while only 8.3% (35/421) were from rural areas. The GPA score of 88.5% of the respondents was 3.75 or higher; 43.5% (183) had scores of 4.5–5, and 45.1% (190) had scores of 3.75–4.49. The number of participants that attended governmental high schools was almost equal to the number of participants who attended private high schools. Most of the participants’ fathers were either post-graduates (47.7%) or graduates (32.8%), while the participants’ mothers were either graduates (38.7%) or completed primary or secondary education (30.6%). Most of the participants (91.4%) were living with their family members. Most of the participants (96.9%) stated that they joined the medical field by choice, and after beginning study, most of them showed continued interest in medicine (91.2%).

**Table 1:** Numbers and percentages of the participants who have ever cheated and the study variables.

Characteristics	Participants	Ever cheated			
	n(%)	No, n (%)	Yes, n (%)	OR (95% CI)	p-value
Gender					
Male	266(63.2)	175 (65.8)	91 (34.2)	1.92(1.21–3.04)	0.005
Female	155(36.8)	122 (78.7)	33 (21.3)	1	
Original background					
Urban	386(91.7)	274 (71.0)	112 (29.0)	1	
Rural	35(8.3)	23 (65.7)	12 (34.3)	1.28(0.61–2.65)	0.513
Grade (GPA)					
4.50–5	183(43.5)	142 (77.6)	41 (22.4)	1	
3.75–4.49	190(45.1)	129 (67.9)	61 (32.1)	2.93(1.50–5.70)	0.002
< 3.75	48(11.4)	26 (54.2)	22 (45.8)	1.64(1.03–2.60)	0.036
Type of high school attended					
Governmental school	210(49.9)	154 (73.3)	56 (26.6)	1	
Private school	211(50.1)	143 (67.8)	68 (32.2)	1.30(0.86–1.99)	0.211
Mother’s education level					
Illiterate	17(4.1)	13 (76.5)	4 (23.5)	1	
Primary or Secondary education	129(30.6)	94 (72.9)	35 (27.1)	121(0.37–3.96)	0.752
Graduate	163(38.7)	112 (68.7)	51 (31.3)	1.48(0.46–4.76)	0.511
Postgraduate or above	112(26.6)	78 (69.6)	34 (30.4)	1.42(0.43–4.66)	0.566
Father’s education level					
Illiterate	3(0.7)	3 (100)	0 (0.0)	—-	
Primary or Secondary education	79(18.8)	56 (70.9)	23 (29.1)	1.36(0.73–2.55)	0.335
Graduate	138(32.8)	106 (76.8)	32 (23.2)	1	
Postgraduate or above	201(47.7)	132 (65.7)	69 (34.3)	1.73(1.06–2.83)	0.028
Location of student’s residence					
In the university hostel or with friends	36(8.6)	31 (86.1)	5 (13.9)	1	
With family	385(91.4)	266 (69.1)	119 (30.9)	2.77(1.05–7.31)	0.039
Joined medical field of study by your own choice					
Yes	408(96.9)	290 (71.1)	118 (28.9)	1	
No	13(3.1)	7 (53.8)	6 (46.2)	2.10(0.69–6.40)	0.189
Interested in/Like medical field of study					
Yes	384(91.2)	271 (70.6)	113 (29.4)	1	
No	37(8.8)	26 (70.3)	11 (29.7)	1.01(0.48–2.12)	0.969

Of 421 participants, approximately 29% of them confessed to cheating. Among the cheaters, male participants (34.2%; OR = 1.92) were more common compared to female participants (21.3%), and the difference was statistically significant (p = 0.005).

The participants with high GPA scores (4.50–5) were less involved in cheating (22.4%). The participants with lower GPA scores were more involved in cheating activities, and the results were statistically significant (p< 0.05). The participants living with their family members were more likely to cheat (30.9%; OR = 2.77) compared to those living in the university hostel or living with their friends (13.9%), and the results were statistically significant (p = 0.032).

Table 2 shows the responses of the participants to different misconduct scenarios. In the first scenario, a student copies verbatim from the Internet and other published sources (textbooks or papers) and acknowledges the sources in the reference list; 246 (58.4%) participants expressed that the student mentioned in the scenario was wrong, and 203 (48.2%) participants had or would consider doing the same in the future. The second scenario related to copying from the Internet and other published sources (textbooks or research articles) without acknowledging the sources; 391 (92.2%) participants stated that the student in the scenario was wrong, whereas 79 (18.8%) had or would consider doing the same in the future. The summarized data for all the scenarios have been provided in Table 2.

**Table 2:** Responses of the participants to different misconduct scenarios (in number and percentages).

Scenarios	The student is wrong		Have you done or would consider doing the same	
	Yes n (%)	No n (%)	Yes n (%)	No n(%)
For an assignment, a student copies verbatim (word-for-word) from the Internet and other published sources (textbooks, papers) and lists them as references.	246(58.4)	175(41.6)	203(48.2)	218(51.8)
For an assignment, a student copies from the Internet and other published sources (textbooks, papers) without acknowledging the sources.	391(92.2)	30(7.1)	79(18.8)	342(81.2)
For an assignment, a student copies from assignments submitted earlier by senior students.	380(90.3)	41(9.7)	104(24.7)	317(75.3)
A student helps a friend by writing an assignment for him/her.	265(62.9)	156(37.1)	212(50.4)	209(49.6)
A student lends his work to a friend to copy.	357(84.8)	64(15.2)	192(45.6)	229(54.4)
A student copies a friend’s work without telling him.	409(97.1)	12(2.9)	20(4.8)	401(95.2)
A student re-submits the same report for another part of the course.	300(71.3)	121(28.7)	162(38.5)	259(61.5)
While plotting a graph for an experiment, a student omits and/or adds data points to show the desired results.	354(84.1)	67(15.9)	61(14.5)	360(85.5)
A student writes “Examination–normal” in his patient presentation when he has not performed the clinical examination for that patient.	407(96.7)	14(3.3)	107(25.4)	314(74.6)
A student fakes an illness to justify an absence from an educational activity.	404(96.0)	17(4.0)	87(20.7)	334(79.3)
A student submits a fake medical certificate to justify an absence.	405(96.2)	16(3.8)	69(16.4)	352(83.6)
A student forges a professor’s signature on a piece of work, such as a clinical log book	406(96.4)	15(3.6)	116(27.6)	305(72.4)
A student cheats in an examination or helps another student to cheat.	411(97.6)	10(2.4)	69(16.4)	352(83.6)
A student reports that another student was cheating during an examination.	217(51.5)	204(48.5)	40(9.5)	381(90.5)

Table 3 shows the responses of the participants by gender to various scenarios of misconduct. In the scenario where a student copies from the Internet and other published sources (textbooks, research articles) without acknowledging the sources, 391 (92.2%) participants stated that the student in the scenario was wrong, of whom the majority were females 148 (95.4%) compared with the males 243 (91.3%), but the results were not statistically significant (OR = 2.01; p = 0.118). Additionally, 79 (18.8%) participants [61 (22.9%) men and 18 (11.6%) women] had done or would consider doing the same, and the results were statistically significant (OR = 2.26; p = 0.005). In the second scenario, where the student copies from assignments submitted earlier by the senior students, 380 (90.3%) participants [238 (89.5%) men and 142 (91.6%) women] responded that the student in the scenario was wrong. The majority of these respondents were women [142 (91.6%)] compared to men [238 (89.5%)], and the results were not statistically significant (OR = 1.28; p = 0.476). However, 104 (27.4%) participants [80 (30.1%) men and 24 (15.5%) women] have done or would consider doing the same, and the ratio of men was significantly higher than women (OR = 2.35; p = 0.001). The results of all the scenarios discussed with the participants are shown in Table 3.

**Table 3:** Responses of the participants to different misconduct scenarios in relation to their gender.

Scenarios	Gender	The student is wrong.				Have you done or would consider doing the same			
		No[n(%)]	Yes [n(%)]	OR(95%CI)	p	No[n(%)]	Yes [n(%)]	OR(95%CI)	p
For an assignment, a student copies verbatim (word-for-word) from the Internet and other published sources (textbooks, papers) and lists them as references.	Male	109(41)	157(59.0)	1.07(0.71–1.60)	0.748	138(51.9)	128(48.1)	1	
	Female	66(42.6)	89(57.4)	1		80(51.6)	75(48.4)	1.01(0.68–1.50)	0.958
For an assignment, a student copies from the Internet and other published sources (textbooks, papers) without acknowledging the sources.	Male	23(8.6)	243 (91.3)	1		61(22.9)	61 (22.9)	2.26(1.28–3.40)	0.005
	Female	7(4.5)	148 (95.4)	2.01(0.84–4.78)	0.118	137(88.4)	18 (11.6)	1	
For an assignment, a student copies from assignments submitted earlier by senior students.	Male	28(10.5)	238 (89.5)	1		186(69.9)	80 (30.1)	2.35(1.41–3.90)	0.001
	Female	13(8.4)	142 (91.6)	1.28(0.65–2.56)	0.476	131(84.5)	24 (15.5)	1	
A student helps a friend by writing an assignment for him/her.	Male	99(37.2)	167(62.8)	1		130(48.9)	136(51.1)	1.08(0.73–1.61)	0.678
	Female	57(36.8)	98(63.2)	1.09(0.68–1.54)	0.928	79(51.0)	76(49.0)	1	
A student lends his work to a friend to copy.	Male	39(14.7)	227(85.3)	1.12(0.65–1.93)	0.686	136(51.1)	130(51.1)	1.43(0.96–2.14)	0.078
	Female	25(16.1)	130(83.9)	1		93(60.0)	62(40.0)	1	
A student copies a friend’s work without telling him.	Male	10(3.8)	256 (96.2)	1		249(93.6)	17 (6.4)	3.46(0.99–12.0)	0.05
	Female	2(1.3)	153 (98.7)	3.0(0.65–13.82)	0.161	152(98.1)	3 (1.9)	1	
A student re-submits the same report for another part of the course.	Male	73(27.4)	193 (72.5)	1.19(0.77–1.83)	0.441	148(55.6)	118 (44.4)	2.01(1.31–3.07)	0.001
	Female	48(31.0)	107 (69.0)	1		111(71.6)	44 (28.3)	1	
While plotting a graph for an experiment, a student omits and/or adds data points to show the desired results.	Male	49(18.4)	217 (81.6)	1		220(82.7)	46 (17.3)	1.95(1.05–3.62)	0.035
	Female	18(11.6)	137 (88.4)	1.72(0.96–3.07)	0.068	140(90.3)	15 (9.7)	1	
A student writes “Examination–normal” in his patient presentation when he has not performed the clinical examination for that patient.	Male	11(4.1)	225(95.9)	1		198(74.4)	68(26.6)	1.02(0.65–1.61)	0.927
	Female	3(1.9)	152(98.1)	2.19(0.60–7.96)	0.236	116(74.8)	39(25.2)	1	
A student fakes an illness to justify an absence from an educational activity.	Male	10(3.8)	256(96.2)	1.21(0.45–3.25)	0.704	213(80.1)	53(19.9)	1	
	Female	7(4.5)	148(95.5)	1		34(21.9)	34(21.9)	1.13(0.69–1.83)	0.623
A student submits a fake medical certificate to justify an absence.	Male	8(3.0)	258(97.0)	1.75(0.65–4.77)	0.271	224(84.2)	42(15.8)	1	
	Female	8(5.2)	147(94.8)	1		128(82.6)	27(17.4)	1.12(0.66–1.91)	0.663
A student forges a professor’s signature on a piece of work, such as a clinical log book	Male	9(3.4)	257(96.6)	1.15(0.40–3.29)	0.795	197(74.1)	69(25.9)	1	
	Female	6(3.9)	149(96.1)	1		108(69.7)	47(30.3)	1.24(0.80–1.93)	0.332
A student cheats in an examination or helps another student to cheat.	Male	7(2.6)	259 (97.3)	1		210(78.9)	56 (21.1)	2.91(1.53–5.52)	0.00
	Female	3(1.9)	152 (98.1)	1.37(0.35–5.37)	0.652	142(91.6)	13 (8.4)	1	
A student reports that another student was cheating during an examination.	Male	111(41.7)	155 (58.3)	2.09(1.40–3.14)	0.0001	242(91.0)	24 (9.0)	1	
	Female	93(60.0)	62 (40.0)	1		139(89.7)	16 (10.3)	1.16(0.60–2.26)	0.661

**Fig 1:**
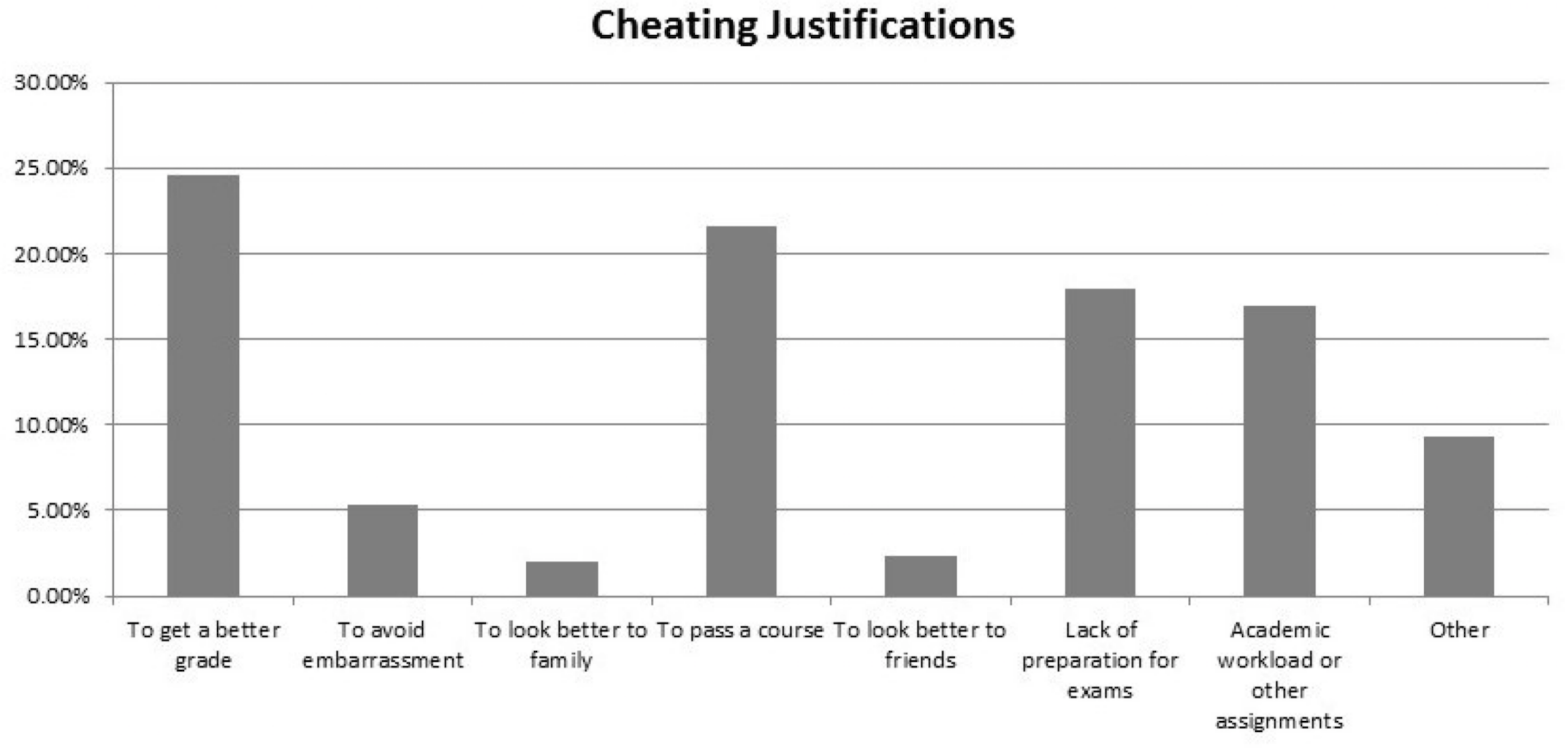
Reasons given by the participating students for cheating.

The responses of the participants to different misconduct scenarios related to their academic grades are given in Table 4. In the scenario where a student copies from the Internet and other published sources (textbooks or research articles) without acknowledging the sources, 391 (92.2%) participants stated that the student in the scenario was wrong [of them, majority with 3.75–4.49 GPA (91.6%, OR = 2.51) and with 4.50–5 GPA (97.3%; OR = 8.21)], and the results were statistically significant (p<0.05). However, 79 (18.8%) participants had done or would consider doing the same [of them majority with < 3.75 GPA (31.2%; OR = 3.33), and with 3.75–4.49 GPA (22.1%; OR = 2.08), compared to the 4.50–5 GPA 22 (12.0%)], and the results were statistically significant (p<0.05). The scenarios and the participants’ responses are shown in Table 4. Fig 1 summarizes the reasons for cheating given by the participating students. The participants who admitted that they have cheated justified their cheating behavior by giving various reasons. Most of the students attributed this act to earning better grades (24.6%) or to passing the course (21.6%) or because they were not fully prepared for the exams (17.9%).

**Table 4:** Responses of the participants to different misconduct scenarios in relation to their academic grades.

Scenarios	Grade (GPA)	The student is wrong				Have you done or would consider doing the same			
		No[n(%)]	Yes[n(%)]	OR(95%CI)	p	No[n(%)]	Yes[n(%)]	OR(95%CI)	p
For an assignment, a student copies verbatim (word-for-word) from the Internet and other published sources (textbooks, papers) and lists them as references	< 3.75	25(52.1)	23(47.9)	1		19(39.6)	29(60.4)	2.01(1.05–3.84)	0.035
	3.75–4.49	77(40.5)	113(59.5)	1.60(0.85–3.01)	0.15	95(50)	95(50.0)	1.32(0.87–1.98)	0.187
	4.50–5	73(39.9)	110(60.1)	1.64(0.86–3.10)	0.13	104(56.8)	79(43.2)	1	
For an assignment, a student copies from the Internet and other published sources (textbooks, papers) without acknowledging the sources	< 3.75	9(18.8)	39(81.2)	1		33(68.8)	15(31.2)	3.33(1.56–7.08)	0.002
	3.75–4.49	16(8.4)	174(91.6)	2.51(1.03–6.09)	0.042	148(77.9)	42(22.1)	2.08(1.18–3.64)	0.011
	4.50–5	5(2.7)	178(97.3)	8.21(2.61–25.86)	0.0001	161(88.0)	22(12.0)	1	
For an assignment, a student copies from assignments submitted earlier by senior students	< 3.75	8(16.7)	40(83.3)	1		35(72.9)	13(27.1)	1.63(0.78–3.40)	0.195
	3.75–4.49	26(13.7)	164(86.3)	1.26(0.53–2.30)	0.598	133(70.0)	57(30.0)	1.88(1.16–3.05)	0.011
	4.50–5	7(3.8)	176(96.2)	5.03(1.72–14.67)	0.003	149(81.4)	34(18.6)	1	
A student helps a friend by writing an assignment for him/her	< 3.75	24(50)	24(50)	1		17(35.4)	31(64.6)	2.02(1.05–3.90)	0.035
	3.75–4.49	76(40)	114(60)	1.50(0.79–2.83)	0.211	100(52.6)	90(47.4)	1	
	4.50–5	56(30.6)	127(69.4)	2.27(1.19–4.33)	0.013	92(50.3)	91(49.7)	1.10(0.73–1.65)	0.649
A student lends his work to a friend to copy	< 3.75	12(25)	36(75)	1		22(45.8)	26(54.2)	1.56(0.82–2.94)	0.173
	3.75–4.49	31(16.3)	159(83.7)	1.71(0.80–3.65)	0.166	108(56.8)	82(43.2)	1	
	4.50–5	21(11.5)	162(88.5)	2.57(1.16–5.70)	0.020	99(54.1)	84(45.9)	1.12(0.74–1.68)	0.594
A student copies a friend’s work without telling him	< 3.75	8(16.7)	40(83.3)	1		43(89.6)	5(10.4)	2.92(0.88–9.66)	0.079
	3.75–4.49	4(2.1)	186(97.9)	9.3(2.67–32.39)	0.0001	182(95.8)	8(4.2)	1.10(0.39–3.11)	0.850
	4.50–5	0(0)	183(100)	0	0	176(96.2)	7(3.8)	1	
A student re-submits the same report for another part of the course	< 3.75	15(31.3)	33(68.8)	1		28(58.3)	20(41.7)	1.36(0.71–2.60)	0.353
	3.75–4.49	58(30.5)	132(69.5)	1.03(0.52–2.05)	0.923	111(58.4)	79(41.6)	1.36(0.89–2.06)	0.155
	4.50–5	48(26.2)	135(73.8)	1.28(0.64–2.56)	0.488	120(65.6)	63(34.4)	1	
While plotting a graph for an experiment, a student omits and/or adds data points to show the desired results	< 3.75	19(39.6)	29(60.4)	1		34(70.8)	14(29.2)	3.78(1.71–8.31)	0.001
	3.75–4.49	36(18.9)	154(81.1)	2.80(1.41–5.55)	0.003	161(84.7)	29(15.3)	1.65(0.88–3.09)	0.117
	4.50–5	12(6.6)	171(93.4)	9.34(4.10–21.26)	0.0001	165(90.2)	18(9.8)	1	
A student writes “Examination–normal” in his patient presentation when he has not performed the clinical examination for that patient	< 3.75	6(12.5)	42(87.5)	1		33(68.8)	15(31.3)	1.57(0.78–3.18)	0.205
	3.75–4.49	5(2.6)	185(97.4)	5.28(1.54–18.14)	0.008	139(73.2)	51(26.8)	1.27(0.79–2.04)	0.321
	4.50–5	3(1.6)	180(98.4)	8.57(2.06–35.68)	0.003	142(77.6)	41(22.4)	1	
A student fakes an illness to justify an absence from an educational activity	< 3.75	6(12.5)	42(87.5)	1		34(70.8)	14(29.2)	1.94(0.94–4.03)	0.075
	3.75–4.49	8(4.2)	182(95.8)	3.25(1.07–9.86)	0.037	149(78.4)	41(21.6)	1.30(0.78–2.17)	0.320
	4.50–5	3(1.6)	180(98.4)	8.57(2.06–35.68)	0.003	151(82.5)	32(17.5)	1	
A student submits a fake medical certificate to justify an absence	< 3.75	4(8.3)	44(91.7)	1		37(77.1)	11(22.9)	1.65(0.75–3.60)	0.213
	3.75–4.49	7(3.7)	183(96.3)	2.38(0.67–8.48)	0.182	160(84.2)	30(15.8)	1.04(0.59–1.81)	0.896
	4.50–5	5(2.7)	178(97.3)	3.24(0.83–12.55)	0.089	155(84.7)	28(15.3)	1	
A student forges a professor’s signature on a piece of work, such as a clinical log book	< 3.75	4(8.3)	44(91.7)	1		34(70.8)	14(29.2)	1.32(0.65–2.69)	0.433
	3.75–4.49	7(3.7)	183(96.3)	2.38(0.67–8.48)	0.182	145(76.3)	45(23.7)	1	
	4.50–5	4(2.2)	179(97.8)	4.07(0.98–16.91)	0.054	126(68.9)	57(31.1)	1.46(0.92–2.30)	0.107
A student cheats in an examination or helps another student to cheat	< 3.75	4(8.3)	44(91.7)	1		32(66.7)	16(33.3)	3.48(1.66–7.30)	0.001
	3.75–4.49	5(2.6)	185(97.4)	3.36(0.87–13.04)	0.079	160(84.2)	30(15.8)	1.30(0.73–2.34)	0.374
	4.50–5	1(0.5)	182(99.5)	16.54(1.80–151.71)	0.013	160(87.4)	23(12.6)	1	
A student reports that another student was cheating during an examination	< 3.75	21(43.8)	27(56.3)	1.28(0.68–2.43)	0.44	42(87.5)	6(12.5)	2.23(0.78–6.38)	0.134
	3.75–4.49	95(50)	95(50)	1		167(87.9)	23(12.1)	2.15(1.02–4.56)	0.045
	4.50–5	88(48.1)	95(51.9)	1.08(0.72–1.62)	0.712	172(94.0)	11(6.0)	1	

## Discussion

To the best of our knowledge, this is the first study conducted at a medical school in Saudi Arabia to investigate this important and sensitive issue of cheating. In the current study, we found that cheating behavior is prevalent among Saudi medical students, similar to the medical students/colleges of other countries, but it is lower in comparison with the previously published reports [12,13,25]. Approximately 29% of the participants in this study confessed that they had cheated during their academic studies, which is less compared with earlier reports showing 25–35% self-reported cheating and up to 90% self-reported plagiarism [13,23].

Cheating can have negative impacts on honesty in the workplace and the quality of the healthcare system [26,27]. This study investigated the sensitive topic of cheating. The study is unique as it was conducted in the conservative and religious country of Saudi Arabia, which encourages and calls for honesty and ethically perfect behaviors following Islamic Shari’ah law. Considering the context mentioned above, the prevalence of cheating should be less common. However, based on the available medical services and clinical expertise present in the country, it seems that there is an under-reporting of misconduct. Therefore, the present study was conducted to determine the prevalence of cheating and to assess the attitude and the justification for such misconduct.

In this study, male students were found to be more involved in cheating than female students. This finding is supported by three other studies conducted elsewhere [12,13,14]. In contrast, Dundee Medical School reported no significant gender difference between the responses of male and female students [17]. Female students may be apprehensive of being caught as cheaters and afraid of its consequences on social stigma, while male students seem to be more nonchalant, careless or bold about cheating regardless of the consequences that they might face if caught.

We found an inverse relationship between the students’ GPA score and the prevalence of cheating behavior. The higher GPA students were less involved in such misconduct than those with lower GPA scores. This finding could be explained by the assumption that a student achieving a low GPA score wants to pass the exam by any means. In contrast, the students scoring a high GPA depend on their study for the right answer because of their hard work, self-confidence, and knowledge that the attempted cheating of lazy students is useless or unethical. This finding was supported by the previous study performed with pharmacy students [14]. However, another study did not find any significant difference in committing academic misconduct between high and low GPA scoring students [5].

In the present study, the students that lived in the university hostel or with their friends were less likely to be involved in cheating (1 out of 7 students) compared to those who lived with their families (1 out of 3 students). An explanation may be that the students living outside, that is, in a university hostel or with friends, have more free time to study than those living with their families because of family-related events, social obligations and cultural responsibilities.

The cheating behavior was considered wrong by almost all the students during the examination (97.7%). However, around 90% of the participants ignored cheating by others during exams, despite the fact that approximately 51% of them considered ‘reporting’ to be a good practice. This behavior is supported by similar findings in a previously published study from the USA [4].

Surprisingly, in this study, ≥27% of the participants stated that they had or would consider forging a professor’s signature on a piece of work. A study conducted in Pakistan demonstrated a lower percentage of such misconduct [6]. This finding may be explained because of fewer strict regulations or punishment in the higher education system in Saudi Arabia. Therefore, implementing stringent rules and punishments is warranted. Additionally, sets of values and attitudes of the students require more exploration and attention to correct the erroneous attitudes. The majority of the students in our study justified the act of cheating for reasons of helping their friends, getting better grades, passing the course or lacking proper preparation for the examinations.

Academic misconduct must be taken seriously, and following several years of punitive measures, perhaps it is now time to improve the sense of personal reward and moral identity related to academic integrity [28]. As a preventive measure, some studies suggested that students need awareness and training to avoid plagiarism behavior [29,30]. Likewise, another study suggested that educators around the world must address cheating in their institutions by conducting in-depth research on effective ways to reduce its prevalence [31]. Based on the findings, we think that students must be aware of the effects of cheating on their future careers and ultimately the quality of the healthcare they will provide to their community, nation or humanity. As a remedial measure, some recently introduced courses in the medical degree, such as ‘professionalism’ and ‘medical ethics,’ need to be reviewed and updated to yield better outcomes regarding behavioral change. Additionally, religious inclination and practice might help to prevent academic misconduct, as religious belief is one of the most influential forces to control any act, thought, mental status or emotional event. Being one of the most religious states (as Islamic Shari’ah law is strictly practiced in Saudi Arabia) in the world, the religious strength of Islamic beliefs can be exploited for creating awareness among students to prevent cheating.

Our study highlights the prevalence and other predisposing factors that can lead to cheating among medical students and suggests preventive measures. A study conducted in India considered punishment options that can be applied for academic defaulters and suggested various categories of punishments to control this behavior [20]. Unfortunately, no strict rules or punishment options have been suggested or implemented or are available as a preventive measure in Saudi Arabia. However, an earlier study published in Greece suggested that teaching the academic honor code may reduce cheating if appropriately applied [18].

Despite the significant findings generated from this study, it is important to mention the limitations of the present study. For example, (i) various types of biases existed during the study, that is, response bias and observer effects, (ii) we failed to explore the difference between the genders showing an increased willingness to report cheating or other academic misconduct, and (iii) the applied analysis lacks explanation of confounding variables, for example, students living away from home are generally males rather than females, and this might affect the impact of location of residence as a factor. Currently, our group and collaborators are planning future studies to assess the behavioral change outcomes of the students who take well-designed courses on medical ethics, professionalism, and honor codes and to study the behaviors of non-compliant students and their reasoning.

## Conclusion

In conclusion, we observed a high prevalence of cheating behavior among the Saudi medical students, predominantly in males, and they deliberately justify their misconduct with strange reasons; this finding is alarming in a reputable governmental institution and warrants implementation of some stringent preventive measures. Although some participants tried to justify their behavior, they still considered it a mistake or misconduct. However, as a preventive strategy, the implementation of strict punishments, introduction of ethical courses, implementation of a code of conduct, educating students on cheating and plagiarism through workshops or other means, and the creation of awareness about ethical practices among medical students by harnessing the potential of Islamic religious belief might be helpful to prevent cheating.

## Supporting information

S1 Appendix Survey questionnaire used in this study. (DOCX)

S1 Datapoints Processed datapoints used during the study. (PDF)
