# Correction: Self-reported cheating among medical students: An alarming finding in a cross-sectional study from Saudi Arabia

**DOI:** 10.1371/journal.pone.0215862

**Published:** 2019-04-18

**Authors:** Hamza Mohammad Abdulghani, Shafiul Haque, Yousef Abdullah Almusalam, Saleh Lafi Alanezi, Yazeed Abdulaziz Alsulaiman, Mohammad Irshad, Nehal Khamis

The authors amend the title of this article [1] to align with the project title as approved by the IRB and to more accurately reflect the content of the study which addressed misconduct issues including but not limited to cheating. The updated title is:

Academic misconduct among medical students: A cross-sectional study.

The author byline is incorrect. The correct byline is:

Hamza Mohammad Abdulghani, Shafiul Haque, Yousef Abdullah Almusalam, Saleh Lafi Alanezi, Yazeed Abdulaziz Alsulaiman, Mohammad Irshad, Nehal Khamis.

The correct citation is:

Abdulghani HM, Haque S, Almusalam YA, Alanezi SL, Alsulaiman YA, Irshad M, et al. (2018) Academic misconduct among medical students: A cross-sectional study. PLoS ONE 13(3): e0194963. https://doi.org/10.1371/journal.pone.0194963

In the Abstract, the penultimate sentence is incorrect. The correct sentences are: In comparison with the previous reports published from different parts of the world, the overall academic misconduct behavior found in Saudi medical students was quite less. The extent of academic misconduct reported as done or considered by students might be considered alarming for any reputable institution.

The following is hereby added to the Statistical Analysis subsection of the Methods: Using SPSS, the Odds ratios (OR) were computed by the logistic regression analysis for the categorical variables of interest without covariance.

The authors also provide the following updates to the Results section:

- In the Results section, there is an error in the second sentence of the third paragraph. The correct sentence is: The logistic regression analysis revealed that among the cheaters, male participants (34.2%; OR = 1.92) were more common compared to female participants (21.3%), and the difference was statistically significant (p = 0.005).
- In the Results section, there are errors in the second and third sentence of the sixth paragraph. The correct sentences are: In the scenario where a student copies from the Internet and other published sources (textbooks, research articles) without acknowledging the sources, 391 (92.2%) participants stated that the student in the scenario was wrong, of whom the majority were females 148 (95.4%) compared with the males 243 (91.3%). The logistic regression analysis results showed high OR (2.01) for female students, but finding was not statistically significant (p = 0.118). Additionally, 79 (18.8%) participants [61 (22.9%) men and 18 (11.6%) women] had done or would consider doing the same, and the results were statistically significant (OR = 2.26; p = 0.005).
- In the Results section, there are errors in the second and third sentences of the seventh paragraph. The correct sentences are: In the scenario where a student copies from the Internet and other published sources (textbooks or research articles) without acknowledging the sources, 391 (92.2%) participants stated that the student in the scenario was wrong. The logistic regression analysis results showed high odd ratio for 4.5–5 GPA (97.3%; OR = 8.21) and 3.75–4.49 GPA (91.6%; OR = 2.51) and the results were statistically significant (p<0.005).

The p value reported in Table 3 for Male respondents on the penultimate survey item (“A student cheats in an examination or helps another student to cheat”) should be reported as p = 0.001.

In addition, the authors provide the following clarifications:

- There are several instances in the article, including in the Abstract, Introduction, Results, and Discussion, where references to “cheating” ought to have instead referred to “academic misconduct”; the text and results pertain to misconduct issues including but not limited to cheating.
- In the Results section describing findings in Table 3, sentences comparing results for females versus males should have referred to “higher proportions” rather than “the majority”.
- The reported misconduct levels reflect behavior not only during medical school but rather over the participant’s full history.

The individual named in the Acknowledgements has clarified that he did not provide permission to be named. As such, the second sentence of the Acknowledgements section is hereby removed. The correct section is: The authors are grateful to all the students who participated in this study.

Individual-level data supporting this study are provided in S1 File with this Correction.

## Supporting information

S1 File Individual-level datapoints used during the study. (XLSX)
