# Supplementary material for: Self-reported cheating among medical students: An alarming finding in a cross-sectional study from Saudi Arabia
Source: PLoS One. 2018 Mar 29;13(3):e0194963. doi: 10.1371/journal.pone.0194963 (PMC5875787; doi:10.1371/journal.pone.0194963)
Supplement: S1 Appendix — (DOCX) [file pone.0194963.s001.docx]

**SI Appendix**

**Survey Questionnaire**

**Personal Data:**

Year of study: Fifth  internship 

Gender: Male  Female 

GPA: <3.5  3.5 -4.5  > 4.5 

Age: ………………

**Social predictors:**

Original background

- Urban
- Rural

Type of high school attended

- Public school
- Private school

Mother’s education level

- Illiterate
- Primary education
- Secondary education
- Graduate
- Postgraduate or above

Father’s education level

- Illiterate
- Primary education
- Secondary education
- Graduate
- Postgraduate or above

Location of student’s residence

- In the university dormitory/hostel
- With family

Joined medical field of study by choice

- Yes
- No

Interest on/Like medical field of study

- Yes
- No

Cheated at high school

- No
- Yes

**Scenarios related to plagiarism**

**For an assignment, a student copies verbatim (word-for-word) from the internet and other published sources (textbooks, papers) and lists them as references.**

1. The student is wrong.

2. Have done or would consider doing the same.

**For an assignment, a student copies from the internet and other published sources (textbooks, papers) without acknowledging the sources.**

1. The student is wrong.

2. Have done or would consider doing the same.

**For an assignment, a student copies from assignments submitted earlier by senior peers.**

1. The student is wrong.

2. Have done or would consider doing the same.

**A student helps a friend by writing an assignment for him/her.**

1. The student is wrong.

2. Have done or would consider doing the same.

**A student lends his work to a friend to copy.**

1. The student is wrong.

2. Have done or would consider doing the same.

**A student copies a friend’s work without telling him.**

1. The student is wrong.

2. Have done or would consider doing the same.

**A student re-submits the same report for another part of the course.**

1. The student is wrong.

2. Have done or would consider doing the same.

**Scenarios related to lying**

**While plotting a graph for an experiment, a student omits and/or adds data points to show the desired results.**

1. The student is wrong.

2. Have done or would consider doing the same.

**A student writes “Examination – normal” in his patient presentation when he has not performed the procedure.**

1. The student is wrong.

2. Have done or would consider doing the same.

**A student fakes an illness to justify an absence.**

1. The student is wrong.

2. Have done or would consider doing the same.

**A student submits a fake medical certificate to justify an absence.**

1. The student is wrong.

2. Have done or would consider doing the same.

**A student forges a professor’s signature on a piece of work.**

1. The student is wrong.

2. Have done or would consider doing the same.

**Scenarios related to cheating**

**A student cheats in an examination.**

1. The student is wrong.

2. Have done or would consider doing the same.

**A student reports that another student was cheating during an examination.**

1. The student is wrong.

2. Have done or would consider doing the same.

**A model goes missing from the Anatomy lab and a student who is aware of the culprit reports the information to the concerned faculty/staff.**

1. The student is wrong.

2. Have done or would consider doing the same.

**Justification of cheating:**

1 To get a better grade

2 To look better to family

3 To look better to friends

4 To avoid embarrassment

5 To pass a course

6 Lack of preparation for exams

7 Academic workload or other assignments
