# Supplementary material for: Self-reported cheating among medical students: An alarming finding in a cross-sectional study from Saudi Arabia
Source: PLoS One. 2018 Mar 29;13(3):e0194963. doi: 10.1371/journal.pone.0194963 (PMC5875787; doi:10.1371/journal.pone.0194963)
Supplement: S1 Datapoints — (PDF) [file pone.0194963.s002.pdf]

Frequencies

Notes

Output Created		02-Nov-2015 11:28:43
Comments		
Input	Data	H:\Hamza (Cheating project)\Cheating data(16102015).sav
	Active Dataset	DataSet2
	Filter	<none>
	Weight	<none>
	Split File	<none>
	N of Rows in Working Data File	421
Missing Value Handling	Definition of Missing	User-defined missing values are treated as missing.
	Cases Used	Statistics are based on all cases with valid data.
Syntax		FREQUENCIES VARIABLES=SC1 SCC1 SC2 SCC2 SC3 SCC3 SC4 SCC4 SC5 SCC5 SC6 SCC6 SC7 SCC7 SC8 SCC8 SC9 SCC9 SC10 SCC10 SC11 SCC11 SC12 SCC12 SC13 SCC13 SC14 SCC14 /ORDER=ANALYSIS.
Resources	Processor Time	00 00:00:00.000
	Elapsed Time	00 00:00:00.010

```
[DataSet2] H:\Hamza (Cheating project)\Cheating data(16102015).sav
```

Frequency Table

SC1

		Frequency	Percent	Valid Percent	Cumulative Percent
Valid	Yes	243	57.7	57.7	57.7
	No	178	42.3	42.3	100.0
	Total	421	100.0	100.0	

SCC1

		Frequency	Percent	Valid Percent	Cumulative Percent
Valid	Yes	203	48.2	48.2	48.2
	No	218	51.8	51.8	100.0
	Total	421	100.0	100.0	

SC2

		Frequency	Percent	Valid Percent	Cumulative Percent
Valid	Yes	391	92.9	92.9	92.9
	No	30	7.1	7.1	100.0
	Total	421	100.0	100.0	

SCC2

		Frequency	Percent	Valid Percent	Cumulative Percent
Valid	Yes	79	18.8	18.8	18.8
	No	342	81.2	81.2	100.0
	Total	421	100.0	100.0	

SC3

		Frequency	Percent	Valid Percent	Cumulative Percent
Valid	Yes	380	90.3	90.3	90.3
	No	41	9.7	9.7	100.0
	Total	421	100.0	100.0	

SCC3

		Frequency	Percent	Valid Percent	Cumulative Percent
Valid	Yes	111	26.4	26.4	26.4
	No	310	73.6	73.6	100.0
	Total	421	100.0	100.0	

SC4

		Frequency	Percent	Valid Percent	Cumulative Percent
Valid	Yes	265	62.9	63.1	63.1
	No	155	36.8	36.9	100.0
	Total	420	99.8	100.0	
Missing	System	1	.2		
Total		421	100.0		

SCC4

		Frequency	Percent	Valid Percent	Cumulative Percent
Valid	Yes	212	50.4	50.4	50.4
	No	209	49.6	49.6	100.0
	Total	421	100.0	100.0	

SC5

		Frequency	Percent	Valid Percent	Cumulative Percent
Valid	Yes	357	84.8	84.8	84.8
	No	64	15.2	15.2	100.0
	Total	421	100.0	100.0	

SCC5

		Frequency	Percent	Valid Percent	Cumulative Percent
Valid	Yes	193	45.8	45.8	45.8
	No	228	54.2	54.2	100.0
	Total	421	100.0	100.0	

SC6

		Frequency	Percent	Valid Percent	Cumulative Percent
Valid	Yes	409	97.1	97.1	97.1
	No	12	2.9	2.9	100.0
	Total	421	100.0	100.0	

SCC6

		Frequency	Percent	Valid Percent	Cumulative Percent
Valid	Yes	20	4.8	4.8	4.8
	No	401	95.2	95.2	100.0
	Total	421	100.0	100.0	

SC7

		Frequency	Percent	Valid Percent	Cumulative Percent
Valid	Yes	300	71.3	71.3	71.3
	No	121	28.7	28.7	100.0
	Total	421	100.0	100.0	

SCC7

		Frequency	Percent	Valid Percent	Cumulative Percent
Valid	Yes	162	38.5	38.5	38.5
	No	259	61.5	61.5	100.0
	Total	421	100.0	100.0	

SC8

		Frequency	Percent	Valid Percent	Cumulative Percent
Valid	Yes	354	84.1	84.1	84.1
	No	67	15.9	15.9	100.0
	Total	421	100.0	100.0	

SCC8

		Frequency	Percent	Valid Percent	Cumulative Percent
Valid	Yes	61	14.5	14.5	14.5
	No	360	85.5	85.5	100.0
	Total	421	100.0	100.0	

SC9

		Frequency	Percent	Valid Percent	Cumulative Percent
Valid	Yes	407	96.7	96.7	96.7
	No	14	3.3	3.3	100.0
	Total	421	100.0	100.0	

SCC9

		Frequency	Percent	Valid Percent	Cumulative Percent
Valid	Yes	107	25.4	25.4	25.4
	No	314	74.6	74.6	100.0
	Total	421	100.0	100.0	

SC10

		Frequency	Percent	Valid Percent	Cumulative Percent
Valid	Yes	404	96.0	96.0	96.0
	No	17	4.0	4.0	100.0
	Total	421	100.0	100.0	

SCC10

		Frequency	Percent	Valid Percent	Cumulative Percent
Valid	Yes	86	20.4	20.4	20.4
	No	335	79.6	79.6	100.0
	Total	421	100.0	100.0	

SC11

		Frequency	Percent	Valid Percent	Cumulative Percent
Valid	Yes	407	96.7	96.7	96.7
	No	14	3.3	3.3	100.0
	Total	421	100.0	100.0	

SCC11

		Frequency	Percent	Valid Percent	Cumulative Percent
Valid	Yes	73	17.3	17.3	17.3
	No	348	82.7	82.7	100.0
	Total	421	100.0	100.0	

SC12

		Frequency	Percent	Valid Percent	Cumulative Percent
Valid	Yes	407	96.7	96.7	96.7
	No	14	3.3	3.3	100.0
	Total	421	100.0	100.0	

SCC12

		Frequency	Percent	Valid Percent	Cumulative Percent
Valid	Yes	116	27.6	27.6	27.6
	No	305	72.4	72.4	100.0
	Total	421	100.0	100.0	

SC13

		Frequency	Percent	Valid Percent	Cumulative Percent
Valid	Yes	411	97.6	97.6	97.6
	No	10	2.4	2.4	100.0
	Total	421	100.0	100.0	

SCC13

		Frequency	Percent	Valid Percent	Cumulative Percent
Valid	Yes	69	16.4	16.4	16.4
	No	352	83.6	83.6	100.0
	Total	421	100.0	100.0	

SC14

		Frequency	Percent	Valid Percent	Cumulative Percent
Valid	Yes	217	51.5	51.5	51.5
	No	204	48.5	48.5	100.0
	Total	421	100.0	100.0	

SCC14

		Frequency	Percent	Valid Percent	Cumulative Percent
Valid	Yes	39	9.3	9.3	9.3
	No	382	90.7	90.7	100.0
	Total	421	100.0	100.0	

```
SAVE OUTFILE='H:\Hamza (Cheating project)\Cheating data(16102015).sav'
 /COMPRESSED.
SORT CASES BY GPA(D).
FREQUENCIES VARIABLES=GPA1
  /ORDER=ANALYSIS.
```

Frequencies

Notes

Output Created		02-Nov-2015 12:09:35
Comments		
Input	Data	H:\Hamza (Cheating project) \Cheating data(16102015).sav
	Active Dataset	DataSet2
	Filter	<none>
	Weight	<none>
	Split File	<none>
	N of Rows in Working Data File	421
Missing Value Handling	Definition of Missing	User-defined missing values are treated as missing.
	Cases Used	Statistics are based on all cases with valid data.
Syntax		FREQUENCIES VARIABLES=GPA1 /ORDER=ANALYSIS.
Resources	Processor Time	00 00:00:00.000
	Elapsed Time	00 00:00:00.060

```
[DataSet2] H:\Hamza (Cheating project)\Cheating data(16102015).sav
```

Statistics

GPA1

N	Valid	421
	Missing	0

GPA1

		Frequency	Percent	Valid Percent	Cumulative Percent
Valid	2.00 to 2.74	2	.5	.5	.5
	2.75 to 3.74	46	10.9	10.9	11.4
	3.75 to 4.49	190	45.1	45.1	56.5
	4.50 to 5.0	183	43.5	43.5	100.0
	Total	421	100.0	100.0	

```
RECODE GPA1 (3=2) (4=3) (1 thru 2=1) INTO GPA2.
EXECUTE.
CROSSTABS
  /TABLES=Gender GPA2 BY SC1 SCC1 SC2 SCC2 SC3 SCC3 SC4 SCC4 SC5 SCC5 SC6 SCC6 SC7 SCC7 SC8 SCC
  /FORMAT=AVALUE TABLES
  /STATISTICS=CHISQ
  /CELLS=COUNT COLUMN
  /COUNT ROUND CELL.
```

Crosstabs

Notes

Output Created		02-Nov-2015 12:11:57
Comments		
Input	Data	H:\Hamza (Cheating project)\Cheating data(16102015).sav
	Active Dataset	DataSet2
	Filter	<none>
	Weight	<none>
	Split File	<none>
	N of Rows in Working Data File	421
Missing Value Handling	Definition of Missing	User-defined missing values are treated as missing.
	Cases Used	Statistics for each table are based on all the cases with valid data in the specified range(s) for all variables in each table.
Syntax		CROSSTABS<br>/TABLES=Gender GPA2 BY SC1 SCC1 SC2 SCC2 SC3 SCC3 SC4 SCC4 SC5 SCC5 SC6 SCC6 SC7 SCC7 SC8 SCC8 SC9 SCC9 SC10 SCC10 SC11 SCC11 SC12 SCC12 SC13 SCC13 SC14 SCC14<br>/FORMAT=AVALUE TABLES<br>/STATISTICS=CHISQ<br>/CELLS=COUNT COLUMN<br>/COUNT ROUND CELL.
Resources	Processor Time	00 00:00:00.062
	Elapsed Time	00 00:00:00.440
	Dimensions Requested	2
	Cells Available	174762

```
[DataSet2] H:\Hamza (Cheating project)\Cheating data(16102015).sav
```

Case Processing Summary

	Cases					
	Valid		Missing		Total	
	N	Percent	N	Percent	N	Percent
Gender * SC1	421	100.0%	0	.0%	421	100.0%
Gender * SCC1	421	100.0%	0	.0%	421	100.0%
Gender * SC2	421	100.0%	0	.0%	421	100.0%
Gender * SCC2	421	100.0%	0	.0%	421	100.0%
Gender * SC3	421	100.0%	0	.0%	421	100.0%
Gender * SCC3	421	100.0%	0	.0%	421	100.0%
Gender * SC4	421	100.0%	0	.0%	421	100.0%
Gender * SCC4	421	100.0%	0	.0%	421	100.0%
Gender * SC5	421	100.0%	0	.0%	421	100.0%
Gender * SCC5	421	100.0%	0	.0%	421	100.0%
Gender * SC6	421	100.0%	0	.0%	421	100.0%
Gender * SCC6	421	100.0%	0	.0%	421	100.0%
Gender * SC7	421	100.0%	0	.0%	421	100.0%
Gender * SCC7	421	100.0%	0	.0%	421	100.0%
Gender * SC8	421	100.0%	0	.0%	421	100.0%
Gender * SCC8	421	100.0%	0	.0%	421	100.0%
Gender * SC9	421	100.0%	0	.0%	421	100.0%
Gender * SCC9	421	100.0%	0	.0%	421	100.0%
Gender * SC10	421	100.0%	0	.0%	421	100.0%
Gender * SCC10	421	100.0%	0	.0%	421	100.0%
Gender * SC11	421	100.0%	0	.0%	421	100.0%
Gender * SCC11	421	100.0%	0	.0%	421	100.0%
Gender * SC12	421	100.0%	0	.0%	421	100.0%
Gender * SCC12	421	100.0%	0	.0%	421	100.0%
Gender * SC13	421	100.0%	0	.0%	421	100.0%
Gender * SCC13	421	100.0%	0	.0%	421	100.0%
Gender * SC14	421	100.0%	0	.0%	421	100.0%
Gender * SCC14	421	100.0%	0	.0%	421	100.0%
GPA2 * SC1	421	100.0%	0	.0%	421	100.0%
GPA2 * SCC1	421	100.0%	0	.0%	421	100.0%
GPA2 * SC2	421	100.0%	0	.0%	421	100.0%
GPA2 * SCC2	421	100.0%	0	.0%	421	100.0%
GPA2 * SC3	421	100.0%	0	.0%	421	100.0%
GPA2 * SCC3	421	100.0%	0	.0%	421	100.0%

Case Processing Summary

	Cases					
	Valid		Missing		Total	
	N	Percent	N	Percent	N	Percent
GPA2 * SC4	421	100.0%	0	.0%	421	100.0%
GPA2 * SCC4	421	100.0%	0	.0%	421	100.0%
GPA2 * SC5	421	100.0%	0	.0%	421	100.0%
GPA2 * SCC5	421	100.0%	0	.0%	421	100.0%
GPA2 * SC6	421	100.0%	0	.0%	421	100.0%
GPA2 * SCC6	421	100.0%	0	.0%	421	100.0%
GPA2 * SC7	421	100.0%	0	.0%	421	100.0%
GPA2 * SCC7	421	100.0%	0	.0%	421	100.0%
GPA2 * SC8	421	100.0%	0	.0%	421	100.0%
GPA2 * SCC8	421	100.0%	0	.0%	421	100.0%
GPA2 * SC9	421	100.0%	0	.0%	421	100.0%
GPA2 * SCC9	421	100.0%	0	.0%	421	100.0%
GPA2 * SC10	421	100.0%	0	.0%	421	100.0%
GPA2 * SCC10	421	100.0%	0	.0%	421	100.0%
GPA2 * SC11	421	100.0%	0	.0%	421	100.0%
GPA2 * SCC11	421	100.0%	0	.0%	421	100.0%
GPA2 * SC12	421	100.0%	0	.0%	421	100.0%
GPA2 * SCC12	421	100.0%	0	.0%	421	100.0%
GPA2 * SC13	421	100.0%	0	.0%	421	100.0%
GPA2 * SCC13	421	100.0%	0	.0%	421	100.0%
GPA2 * SC14	421	100.0%	0	.0%	421	100.0%
GPA2 * SCC14	421	100.0%	0	.0%	421	100.0%

Gender * SC1

Crosstab

			SC1		
			Yes	No	Total
Gender	Female	Count	88	67	155
		% within SC1	36.2%	37.6%	36.8%
	Male	Count	155	111	266
		% within SC1	63.8%	62.4%	63.2%
Total		Count	243	178	421
		% within SC1	100.0%	100.0%	100.0%

Chi-Square Tests

	Value	df	Asymp. Sig. (2-sided)	Exact Sig. (2-sided)	Exact Sig. (1-sided)
Pearson Chi-Square	.090[a]	1	.764		
Continuity Correction[b]	.039	1	.843		
Likelihood Ratio	.090	1	.764		
Fisher's Exact Test				.838	.421
N of Valid Cases	421				

a. 0 cells (.0%) have expected count less than 5. The minimum expected count is 65.53.
b. Computed only for a 2x2 table

Gender * SCC1

Crosstab

			SCC1		Total
			Yes	No	
Gender	Female	Count	75	80	155
		% within SCC1	36.9%	36.7%	36.8%
	Male	Count	128	138	266
		% within SCC1	63.1%	63.3%	63.2%
Total		Count	203	218	421
		% within SCC1	100.0%	100.0%	100.0%

Chi-Square Tests

	Value	df	Asymp. Sig. (2-sided)	Exact Sig. (2-sided)	Exact Sig. (1-sided)
Pearson Chi-Square	.003[a]	1	.958		
Continuity Correction[b]	.000	1	1.000		
Likelihood Ratio	.003	1	.958		
Fisher's Exact Test				1.000	.519
N of Valid Cases	421				

a. 0 cells (.0%) have expected count less than 5. The minimum expected count is 74.74.
b. Computed only for a 2x2 table

Gender * SC2

Crosstab

			SC2		Total
			Yes	No	
Gender	Female	Count	148	7	155
		% within SC2	37.9%	23.3%	36.8%
	Male	Count	243	23	266
		% within SC2	62.1%	76.7%	63.2%
Total		Count	391	30	421
		% within SC2	100.0%	100.0%	100.0%

Chi-Square Tests

	Value	df	Asymp. Sig. (2-sided)	Exact Sig. (2-sided)	Exact Sig. (1-sided)
Pearson Chi-Square	2.525[a]	1	.112		
Continuity Correction[b]	1.939	1	.164		
Likelihood Ratio	2.692	1	.101		
Fisher's Exact Test				.121	.079
N of Valid Cases	421				

a. 0 cells (.0%) have expected count less than 5. The minimum expected count is 11.05.
b. Computed only for a 2x2 table

Gender * SCC2

Crosstab

			SCC2		Total
			Yes	No	
Gender	Female	Count	18	137	155
		% within SCC2	22.8%	40.1%	36.8%
	Male	Count	61	205	266
		% within SCC2	77.2%	59.9%	63.2%
Total		Count	79	342	421
		% within SCC2	100.0%	100.0%	100.0%

Chi-Square Tests

	Value	df	Asymp. Sig. (2-sided)	Exact Sig. (2-sided)	Exact Sig. (1-sided)
Pearson Chi-Square	8.232[a]	1	.004		
Continuity Correction[b]	7.506	1	.006		
Likelihood Ratio	8.721	1	.003		
Fisher's Exact Test				.004	.003
N of Valid Cases	421				

a. 0 cells (.0%) have expected count less than 5. The minimum expected count is 29.09.
b. Computed only for a 2x2 table

Gender * SC3

Crosstab

			SC3		Total
			Yes	No	
Gender	Female	Count	142	13	155
		% within SC3	37.4%	31.7%	36.8%
	Male	Count	238	28	266
		% within SC3	62.6%	68.3%	63.2%
Total		Count	380	41	421
		% within SC3	100.0%	100.0%	100.0%

Chi-Square Tests

	Value	df	Asymp. Sig. (2-sided)	Exact Sig. (2-sided)	Exact Sig. (1-sided)
Pearson Chi-Square	.510[a]	1	.475		
Continuity Correction[b]	.296	1	.587		
Likelihood Ratio	.520	1	.471		
Fisher's Exact Test				.502	.297
N of Valid Cases	421				

a. 0 cells (.0%) have expected count less than 5. The minimum expected count is 15.10.
b. Computed only for a 2x2 table

Gender * SCC3

Crosstab

			SCC3		Total
			Yes	No	
Gender	Female	Count	26	129	155
		% within SCC3	23.4%	41.6%	36.8%
	Male	Count	85	181	266
		% within SCC3	76.6%	58.4%	63.2%
Total		Count	111	310	421
		% within SCC3	100.0%	100.0%	100.0%

Chi-Square Tests

	Value	df	Asymp. Sig. (2-sided)	Exact Sig. (2-sided)	Exact Sig. (1-sided)
Pearson Chi-Square	11.625[a]	1	.001		
Continuity Correction[b]	10.856	1	.001		
Likelihood Ratio	12.184	1	.000		
Fisher's Exact Test				.001	.000
N of Valid Cases	421				

a. 0 cells (.0%) have expected count less than 5. The minimum expected count is 40.87.
b. Computed only for a 2x2 table

Gender * SC4

Crosstab

			SC4		Total
			Yes	No	
Gender	Female	Count	98	57	155
		% within SC4	37.0%	36.5%	36.8%
	Male	Count	167	99	266
		% within SC4	63.0%	63.5%	63.2%
Total		Count	265	156	421
		% within SC4	100.0%	100.0%	100.0%

Chi-Square Tests

	Value	df	Asymp. Sig. (2-sided)	Exact Sig. (2-sided)	Exact Sig. (1-sided)
Pearson Chi-Square	.008[a]	1	.928		
Continuity Correction[b]	.000	1	1.000		
Likelihood Ratio	.008	1	.928		
Fisher's Exact Test				1.000	.506
N of Valid Cases	421				

a. 0 cells (.0%) have expected count less than 5. The minimum expected count is 57.43.
b. Computed only for a 2x2 table

Gender * SCC4

Crosstab

			SCC4		
			Yes	No	Total
Gender	Female	Count	76	79	155
		% within SCC4	35.8%	37.8%	36.8%
	Male	Count	136	130	266
		% within SCC4	64.2%	62.2%	63.2%
Total		Count	212	209	421
		% within SCC4	100.0%	100.0%	100.0%

Chi-Square Tests

	Value	df	Asymp. Sig. (2-sided)	Exact Sig. (2-sided)	Exact Sig. (1-sided)
Pearson Chi-Square	.172[a]	1	.678		
Continuity Correction[b]	.098	1	.754		
Likelihood Ratio	.172	1	.678		
Fisher's Exact Test				.687	.377
N of Valid Cases	421				

a. 0 cells (.0%) have expected count less than 5. The minimum expected count is 76.95.
b. Computed only for a 2x2 table

Gender * SC5

Crosstab

			SC5		
			Yes	No	Total
Gender	Female	Count	130	25	155
		% within SC5	36.4%	39.1%	36.8%
	Male	Count	227	39	266
		% within SC5	63.6%	60.9%	63.2%
Total		Count	357	64	421
		% within SC5	100.0%	100.0%	100.0%

Chi-Square Tests

	Value	df	Asymp. Sig. (2-sided)	Exact Sig. (2-sided)	Exact Sig. (1-sided)
Pearson Chi-Square	.164[a]	1	.686		
Continuity Correction[b]	.070	1	.792		
Likelihood Ratio	.163	1	.687		
Fisher's Exact Test				.676	.393
N of Valid Cases	421				

a. 0 cells (.0%) have expected count less than 5. The minimum expected count is 23.56.
b. Computed only for a 2x2 table

Gender * SCC5

Crosstab

			SCC5		
			Yes	No	Total
Gender	Female	Count	63	92	155
		% within SCC5	32.6%	40.4%	36.8%
	Male	Count	130	136	266
		% within SCC5	67.4%	59.6%	63.2%
Total		Count	193	228	421
		% within SCC5	100.0%	100.0%	100.0%

Chi-Square Tests

	Value	df	Asymp. Sig. (2-sided)	Exact Sig. (2-sided)	Exact Sig. (1-sided)
Pearson Chi-Square	2.670[a]	1	.102		
Continuity Correction[b]	2.349	1	.125		
Likelihood Ratio	2.680	1	.102		
Fisher's Exact Test				.106	.063
N of Valid Cases	421				

a. 0 cells (.0%) have expected count less than 5. The minimum expected count is 71.06.
b. Computed only for a 2x2 table

Gender * SC6

Crosstab

			SC6		Total
			Yes	No	
Gender	Female	Count	153	2	155
		% within SC6	37.4%	16.7%	36.8%
	Male	Count	256	10	266
		% within SC6	62.6%	83.3%	63.2%
Total		Count	409	12	421
		% within SC6	100.0%	100.0%	100.0%

Chi-Square Tests

	Value	df	Asymp. Sig. (2-sided)	Exact Sig. (2-sided)	Exact Sig. (1-sided)
Pearson Chi-Square	2.156[a]	1	.142		
Continuity Correction[b]	1.357	1	.244		
Likelihood Ratio	2.427	1	.119		
Fisher's Exact Test				.224	.120
N of Valid Cases	421				

a. 1 cells (25.0%) have expected count less than 5. The minimum expected count is 4.42.
b. Computed only for a 2x2 table

Gender * SCC6

Crosstab

			SCC6		Total
			Yes	No	
Gender	Female	Count	3	152	155
		% within SCC6	15.0%	37.9%	36.8%
	Male	Count	17	249	266
		% within SCC6	85.0%	62.1%	63.2%
Total		Count	20	401	421
		% within SCC6	100.0%	100.0%	100.0%

Chi-Square Tests

	Value	df	Asymp. Sig. (2-sided)	Exact Sig. (2-sided)	Exact Sig. (1-sided)
Pearson Chi-Square	4.296[a]	1	.038		
Continuity Correction[b]	3.368	1	.066		
Likelihood Ratio	4.901	1	.027		
Fisher's Exact Test				.055	.028
N of Valid Cases	421				

a. 0 cells (.0%) have expected count less than 5. The minimum expected count is 7.36.
b. Computed only for a 2x2 table

Gender * SC7

Crosstab

			SC7		Total
			Yes	No	
Gender	Female	Count	107	48	155
		% within SC7	35.7%	39.7%	36.8%
	Male	Count	193	73	266
		% within SC7	64.3%	60.3%	63.2%
Total		Count	300	121	421
		% within SC7	100.0%	100.0%	100.0%

Chi-Square Tests

	Value	df	Asymp. Sig. (2-sided)	Exact Sig. (2-sided)	Exact Sig. (1-sided)
Pearson Chi-Square	.594[a]	1	.441		
Continuity Correction[b]	.434	1	.510		
Likelihood Ratio	.590	1	.442		
Fisher's Exact Test				.503	.254
N of Valid Cases	421				

a. 0 cells (.0%) have expected count less than 5. The minimum expected count is 44.55.
b. Computed only for a 2x2 table

Gender * SCC7

Crosstab

			SCC7		Total
			Yes	No	
Gender	Female	Count	44	111	155
		% within SCC7	27.2%	42.9%	36.8%
	Male	Count	118	148	266
		% within SCC7	72.8%	57.1%	63.2%
Total		Count	162	259	421
		% within SCC7	100.0%	100.0%	100.0%

Chi-Square Tests

	Value	df	Asymp. Sig. (2-sided)	Exact Sig. (2-sided)	Exact Sig. (1-sided)
Pearson Chi-Square	10.556[a]	1	.001		
Continuity Correction[b]	9.892	1	.002		
Likelihood Ratio	10.778	1	.001		
Fisher's Exact Test				.001	.001
N of Valid Cases	421				

a. 0 cells (.0%) have expected count less than 5. The minimum expected count is 59.64.
b. Computed only for a 2x2 table

Gender * SC8

Crosstab

			SC8		Total
			Yes	No	
Gender	Female	Count	137	18	155
		% within SC8	38.7%	26.9%	36.8%
	Male	Count	217	49	266
		% within SC8	61.3%	73.1%	63.2%
Total		Count	354	67	421
		% within SC8	100.0%	100.0%	100.0%

Chi-Square Tests

	Value	df	Asymp. Sig. (2-sided)	Exact Sig. (2-sided)	Exact Sig. (1-sided)
Pearson Chi-Square	3.392[a]	1	.066		
Continuity Correction[b]	2.902	1	.088		
Likelihood Ratio	3.526	1	.060		
Fisher's Exact Test				.073	.042
N of Valid Cases	421				

a. 0 cells (.0%) have expected count less than 5. The minimum expected count is 24.67.
b. Computed only for a 2x2 table

Gender * SCC8

Crosstab

			SCC8		Total
			Yes	No	
Gender	Female	Count	15	140	155
		% within SCC8	24.6%	38.9%	36.8%
	Male	Count	46	220	266
		% within SCC8	75.4%	61.1%	63.2%
Total		Count	61	360	421
		% within SCC8	100.0%	100.0%	100.0%

Chi-Square Tests

	Value	df	Asymp. Sig. (2-sided)	Exact Sig. (2-sided)	Exact Sig. (1-sided)
Pearson Chi-Square	4.585[a]	1	.032		
Continuity Correction[b]	3.990	1	.046		
Likelihood Ratio	4.826	1	.028		
Fisher's Exact Test				.032	.021
N of Valid Cases	421				

a. 0 cells (.0%) have expected count less than 5. The minimum expected count is 22.46.
b. Computed only for a 2x2 table

Gender * SC9

Crosstab

			SC9		Total
			Yes	No	
Gender	Female	Count	152	3	155
		% within SC9	37.3%	21.4%	36.8%
	Male	Count	255	11	266
		% within SC9	62.7%	78.6%	63.2%
Total		Count	407	14	421
		% within SC9	100.0%	100.0%	100.0%

Chi-Square Tests

	Value	df	Asymp. Sig. (2-sided)	Exact Sig. (2-sided)	Exact Sig. (1-sided)
Pearson Chi-Square	1.474[a]	1	.225		
Continuity Correction[b]	.869	1	.351		
Likelihood Ratio	1.597	1	.206		
Fisher's Exact Test				.272	.177
N of Valid Cases	421				

a. 0 cells (.0%) have expected count less than 5. The minimum expected count is 5.15.
b. Computed only for a 2x2 table

Gender * SCC9

Crosstab

			SCC9		Total
			Yes	No	
Gender	Female	Count	39	116	155
		% within SCC9	36.4%	36.9%	36.8%
	Male	Count	68	198	266
		% within SCC9	63.6%	63.1%	63.2%
Total		Count	107	314	421
		% within SCC9	100.0%	100.0%	100.0%

Chi-Square Tests

	Value	df	Asymp. Sig. (2-sided)	Exact Sig. (2-sided)	Exact Sig. (1-sided)
Pearson Chi-Square	.008[a]	1	.927		
Continuity Correction[b]	.000	1	1.000		
Likelihood Ratio	.008	1	.927		
Fisher's Exact Test				1.000	.512
N of Valid Cases	421				

a. 0 cells (.0%) have expected count less than 5. The minimum expected count is 39.39.
b. Computed only for a 2x2 table

Gender * SC10

Crosstab

			SC10		Total
			Yes	No	
Gender	Female	Count	148	7	155
		% within SC10	36.6%	41.2%	36.8%
	Male	Count	256	10	266
		% within SC10	63.4%	58.8%	63.2%
Total		Count	404	17	421
		% within SC10	100.0%	100.0%	100.0%

Chi-Square Tests

	Value	df	Asymp. Sig. (2-sided)	Exact Sig. (2-sided)	Exact Sig. (1-sided)
Pearson Chi-Square	.145[a]	1	.704		
Continuity Correction[b]	.015	1	.902		
Likelihood Ratio	.143	1	.706		
Fisher's Exact Test				.799	.443
N of Valid Cases	421				

a. 0 cells (.0%) have expected count less than 5. The minimum expected count is 6.26.
b. Computed only for a 2x2 table

Gender * SCC10

Crosstab

			SCC10		
			Yes	No	Total
Gender	Female	Count	34	121	155
		% within SCC10	39.5%	36.1%	36.8%
	Male	Count	52	214	266
		% within SCC10	60.5%	63.9%	63.2%
Total		Count	86	335	421
		% within SCC10	100.0%	100.0%	100.0%

Chi-Square Tests

	Value	df	Asymp. Sig. (2-sided)	Exact Sig. (2-sided)	Exact Sig. (1-sided)
Pearson Chi-Square	.343[a]	1	.558		
Continuity Correction[b]	.212	1	.645		
Likelihood Ratio	.341	1	.559		
Fisher's Exact Test				.616	.321
N of Valid Cases	421				

a. 0 cells (.0%) have expected count less than 5. The minimum expected count is 31.66.
b. Computed only for a 2x2 table

Gender * SC11

Crosstab

			SC11		Total
			Yes	No	
Gender	Female	Count	147	8	155
		% within SC11	36.1%	57.1%	36.8%
	Male	Count	260	6	266
		% within SC11	63.9%	42.9%	63.2%
Total		Count	407	14	421
		% within SC11	100.0%	100.0%	100.0%

Chi-Square Tests

	Value	df	Asymp. Sig. (2-sided)	Exact Sig. (2-sided)	Exact Sig. (1-sided)
Pearson Chi-Square	2.572[a]	1	.109		
Continuity Correction[b]	1.748	1	.186		
Likelihood Ratio	2.461	1	.117		
Fisher's Exact Test				.156	.095
N of Valid Cases	421				

a. 0 cells (.0%) have expected count less than 5. The minimum expected count is 5.15.
b. Computed only for a 2x2 table

Gender * SCC11

Crosstab

			SCC11		Total
			Yes	No	
Gender	Female	Count	29	126	155
		% within SCC11	39.7%	36.2%	36.8%
	Male	Count	44	222	266
		% within SCC11	60.3%	63.8%	63.2%
Total		Count	73	348	421
		% within SCC11	100.0%	100.0%	100.0%

Chi-Square Tests

	Value	df	Asymp. Sig. (2-sided)	Exact Sig. (2-sided)	Exact Sig. (1-sided)
Pearson Chi-Square	.321[a]	1	.571		
Continuity Correction[b]	.188	1	.665		
Likelihood Ratio	.319	1	.572		
Fisher's Exact Test				.595	.330
N of Valid Cases	421				

a. 0 cells (.0%) have expected count less than 5. The minimum expected count is 26.88.
b. Computed only for a 2x2 table

Gender * SC12

Crosstab

			SC12		Total
			Yes	No	
Gender	Female	Count	149	6	155
		% within SC12	36.6%	42.9%	36.8%
	Male	Count	258	8	266
		% within SC12	63.4%	57.1%	63.2%
Total		Count	407	14	421
		% within SC12	100.0%	100.0%	100.0%

Chi-Square Tests

	Value	df	Asymp. Sig. (2-sided)	Exact Sig. (2-sided)	Exact Sig. (1-sided)
Pearson Chi-Square	.227[a]	1	.634		
Continuity Correction[b]	.038	1	.846		
Likelihood Ratio	.223	1	.637		
Fisher's Exact Test				.779	.414
N of Valid Cases	421				

a. 0 cells (.0%) have expected count less than 5. The minimum expected count is 5.15.
b. Computed only for a 2x2 table

Gender * SCC12

Crosstab

			SCC12		Total
			Yes	No	
Gender	Female	Count	47	108	155
		% within SCC12	40.5%	35.4%	36.8%
	Male	Count	69	197	266
		% within SCC12	59.5%	64.6%	63.2%
Total		Count	116	305	421
		% within SCC12	100.0%	100.0%	100.0%

Chi-Square Tests

	Value	df	Asymp. Sig. (2-sided)	Exact Sig. (2-sided)	Exact Sig. (1-sided)
Pearson Chi-Square	.942[a]	1	.332		
Continuity Correction[b]	.736	1	.391		
Likelihood Ratio	.935	1	.334		
Fisher's Exact Test				.366	.195
N of Valid Cases	421				

a. 0 cells (.0%) have expected count less than 5. The minimum expected count is 42.71.
b. Computed only for a 2x2 table

Gender * SC13

Crosstab

			SC13		Total
			Yes	No	
Gender	Female	Count	152	3	155
		% within SC13	37.0%	30.0%	36.8%
	Male	Count	259	7	266
		% within SC13	63.0%	70.0%	63.2%
Total		Count	411	10	421
		% within SC13	100.0%	100.0%	100.0%

Chi-Square Tests

	Value	df	Asymp. Sig. (2-sided)	Exact Sig. (2-sided)	Exact Sig. (1-sided)
Pearson Chi-Square	.205[a]	1	.651		
Continuity Correction [b]	.015	1	.904		
Likelihood Ratio	.211	1	.646		
Fisher's Exact Test				.752	.464
N of Valid Cases	421				

a. 1 cells (25.0%) have expected count less than 5. The minimum expected count is 3.68.
b. Computed only for a 2x2 table

Gender * SCC13

Crosstab

			SCC13		Total
			Yes	No	
Gender	Female	Count	13	142	155
		% within SCC13	18.8%	40.3%	36.8%
	Male	Count	56	210	266
		% within SCC13	81.2%	59.7%	63.2%
Total		Count	69	352	421
		% within SCC13	100.0%	100.0%	100.0%

Chi-Square Tests

	Value	df	Asymp. Sig. (2-sided)	Exact Sig. (2-sided)	Exact Sig. (1-sided)
Pearson Chi-Square	11.464[a]	1	.001		
Continuity Correction [b]	10.559	1	.001		
Likelihood Ratio	12.480	1	.000		
Fisher's Exact Test				.001	.000
N of Valid Cases	421				

a. 0 cells (.0%) have expected count less than 5. The minimum expected count is 25.40.
b. Computed only for a 2x2 table

Gender * SC14

Crosstab

			SC14		Total
			Yes	No	
Gender	Female	Count	62	93	155
		% within SC14	28.6%	45.6%	36.8%
	Male	Count	155	111	266
		% within SC14	71.4%	54.4%	63.2%
Total		Count	217	204	421
		% within SC14	100.0%	100.0%	100.0%

Chi-Square Tests

	Value	df	Asymp. Sig. (2-sided)	Exact Sig. (2-sided)	Exact Sig. (1-sided)
Pearson Chi-Square	13.089[a]	1	.000		
Continuity Correction[b]	12.368	1	.000		
Likelihood Ratio	13.152	1	.000		
Fisher's Exact Test				.000	.000
N of Valid Cases	421				

a. 0 cells (.0%) have expected count less than 5. The minimum expected count is 75.11.
b. Computed only for a 2x2 table

Gender * SCC14

Crosstab

			SCC14		Total
			Yes	No	
Gender	Female	Count	15	140	155
		% within SCC14	38.5%	36.6%	36.8%
	Male	Count	24	242	266
		% within SCC14	61.5%	63.4%	63.2%
Total		Count	39	382	421
		% within SCC14	100.0%	100.0%	100.0%

Chi-Square Tests

	Value	df	Asymp. Sig. (2-sided)	Exact Sig. (2-sided)	Exact Sig. (1-sided)
Pearson Chi-Square	.050[a]	1	.823		
Continuity Correction[b]	.002	1	.961		
Likelihood Ratio	.050	1	.824		
Fisher's Exact Test				.862	.475
N of Valid Cases	421				

a. 0 cells (.0%) have expected count less than 5. The minimum expected count is 14.36.
b. Computed only for a 2x2 table

GPA2 * SC1

Crosstab

			SC1		
			Yes	No	Total
GPA2	< 3.75	Count	23	25	48
		% within SC1	9.5%	14.0%	11.4%
	3.75 to 4.49	Count	111	79	190
		% within SC1	45.7%	44.4%	45.1%
	4.50 to 5.0	Count	109	74	183
		% within SC1	44.9%	41.6%	43.5%
Total		Count	243	178	421
		% within SC1	100.0%	100.0%	100.0%

Chi-Square Tests

	Value	df	Asymp. Sig. (2-sided)
Pearson Chi-Square	2.183[a]	2	.336
Likelihood Ratio	2.158	2	.340
Linear-by-Linear Association	1.421	1	.233
N of Valid Cases	421		

a. 0 cells (.0%) have expected count less than 5. The minimum expected count is 20.29.

GPA2 * SCC1

Crosstab

			SCC1		Total
			Yes	No	
GPA2	< 3.75	Count	29	19	48
		% within SCC1	14.3%	8.7%	11.4%
	3.75 to 4.49	Count	95	95	190
		% within SCC1	46.8%	43.6%	45.1%
	4.50 to 5.0	Count	79	104	183
		% within SCC1	38.9%	47.7%	43.5%
Total		Count	203	218	421
		% within SCC1	100.0%	100.0%	100.0%

Chi-Square Tests

	Value	df	Asymp. Sig. (2-sided)
Pearson Chi-Square	4.971[a]	2	.083
Likelihood Ratio	4.990	2	.082
Linear-by-Linear Association	4.850	1	.028
N of Valid Cases	421		

a. 0 cells (.0%) have expected count less than 5. The minimum expected count is 23.14.

GPA2 * SC2

Crosstab

			SC2		Total
			Yes	No	
GPA2	< 3.75	Count	39	9	48
		% within SC2	10.0%	30.0%	11.4%
	3.75 to 4.49	Count	174	16	190
		% within SC2	44.5%	53.3%	45.1%
	4.50 to 5.0	Count	178	5	183
		% within SC2	45.5%	16.7%	43.5%
Total		Count	391	30	421
		% within SC2	100.0%	100.0%	100.0%

Chi-Square Tests

	Value	df	Asymp. Sig. (2-sided)
Pearson Chi-Square	15.619[a]	2	.000
Likelihood Ratio	14.311	2	.001
Linear-by-Linear Association	14.897	1	.000
N of Valid Cases	421		

a. 1 cells (16.7%) have expected count less than 5. The minimum expected count is 3.42.

GPA2 * SCC2

Crosstab

			SCC2		Total
			Yes	No	
GPA2	< 3.75	Count	15	33	48
		% within SCC2	19.0%	9.6%	11.4%
	3.75 to 4.49	Count	42	148	190
		% within SCC2	53.2%	43.3%	45.1%
	4.50 to 5.0	Count	22	161	183
		% within SCC2	27.8%	47.1%	43.5%
Total		Count	79	342	421
		% within SCC2	100.0%	100.0%	100.0%

Chi-Square Tests

	Value	df	Asymp. Sig. (2-sided)
Pearson Chi-Square	11.758[a]	2	.003
Likelihood Ratio	11.705	2	.003
Linear-by-Linear Association	11.718	1	.001
N of Valid Cases	421		

a. 0 cells (.0%) have expected count less than 5. The minimum expected count is 9.01.

GPA2 * SC3

Crosstab

			SC3		Total
			Yes	No	
GPA2	< 3.75	Count	40	8	48
		% within SC3	10.5%	19.5%	11.4%
	3.75 to 4.49	Count	164	26	190
		% within SC3	43.2%	63.4%	45.1%
	4.50 to 5.0	Count	176	7	183
		% within SC3	46.3%	17.1%	43.5%
Total		Count	380	41	421
		% within SC3	100.0%	100.0%	100.0%

Chi-Square Tests

	Value	df	Asymp. Sig. (2-sided)
Pearson Chi-Square	13.266[a]	2	.001
Likelihood Ratio	14.489	2	.001
Linear-by-Linear Association	12.101	1	.001
N of Valid Cases	421		

a. 1 cells (16.7%) have expected count less than 5. The minimum expected count is 4.67.

GPA2 * SCC3

Crosstab

			SCC3		Total
			Yes	No	
GPA2	< 3.75	Count	13	35	48
		% within SCC3	11.7%	11.3%	11.4%
	3.75 to 4.49	Count	59	131	190
		% within SCC3	53.2%	42.3%	45.1%
	4.50 to 5.0	Count	39	144	183
		% within SCC3	35.1%	46.5%	43.5%
Total		Count	111	310	421
		% within SCC3	100.0%	100.0%	100.0%

Chi-Square Tests

	Value	df	Asymp. Sig. (2-sided)
Pearson Chi-Square	4.571[a]	2	.102
Likelihood Ratio	4.608	2	.100
Linear-by-Linear Association	2.520	1	.112
N of Valid Cases	421		

a. 0 cells (.0%) have expected count less than 5. The minimum expected count is 12.66.

GPA2 * SC4

Crosstab

			SC4		Total
			Yes	No	
GPA2	< 3.75	Count	24	24	48
		% within SC4	9.1%	15.4%	11.4%
	3.75 to 4.49	Count	114	76	190
		% within SC4	43.0%	48.7%	45.1%
	4.50 to 5.0	Count	127	56	183
		% within SC4	47.9%	35.9%	43.5%
Total		Count	265	156	421
		% within SC4	100.0%	100.0%	100.0%

Chi-Square Tests

	Value	df	Asymp. Sig. (2-sided)
Pearson Chi-Square	7.423[a]	2	.024
Likelihood Ratio	7.389	2	.025
Linear-by-Linear Association	7.402	1	.007
N of Valid Cases	421		

a. 0 cells (.0%) have expected count less than 5. The minimum expected count is 17.79.

GPA2 * SCC4

Crosstab

			SCC4		Total
			Yes	No	
GPA2	< 3.75	Count	31	17	48
		% within SCC4	14.6%	8.1%	11.4%
	3.75 to 4.49	Count	90	100	190
		% within SCC4	42.5%	47.8%	45.1%
	4.50 to 5.0	Count	91	92	183
		% within SCC4	42.9%	44.0%	43.5%
Total		Count	212	209	421
		% within SCC4	100.0%	100.0%	100.0%

Chi-Square Tests

	Value	df	Asymp. Sig. (2-sided)
Pearson Chi-Square	4.594[a]	2	.101
Likelihood Ratio	4.654	2	.098
Linear-by-Linear Association	1.354	1	.245
N of Valid Cases	421		

a. 0 cells (.0%) have expected count less than 5. The minimum expected count is 23.83.

GPA2 * SC5

Crosstab

			SC5		Total
			Yes	No	
GPA2	< 3.75	Count	36	12	48
		% within SC5	10.1%	18.8%	11.4%
	3.75 to 4.49	Count	159	31	190
		% within SC5	44.5%	48.4%	45.1%
	4.50 to 5.0	Count	162	21	183
		% within SC5	45.4%	32.8%	43.5%
Total		Count	357	64	421
		% within SC5	100.0%	100.0%	100.0%

Chi-Square Tests

	Value	df	Asymp. Sig. (2-sided)
Pearson Chi-Square	5.729[a]	2	.057
Likelihood Ratio	5.401	2	.067
Linear-by-Linear Association	5.474	1	.019
N of Valid Cases	421		

a. 0 cells (.0%) have expected count less than 5. The minimum expected count is 7.30.

GPA2 * SCC5

Crosstab

			SCC5		
			Yes	No	Total
GPA2	< 3.75	Count	26	22	48
		% within SCC5	13.5%	9.6%	11.4%
	3.75 to 4.49	Count	83	107	190
		% within SCC5	43.0%	46.9%	45.1%
	4.50 to 5.0	Count	84	99	183
		% within SCC5	43.5%	43.4%	43.5%
Total		Count	193	228	421
		% within SCC5	100.0%	100.0%	100.0%

Chi-Square Tests

	Value	df	Asymp. Sig. (2-sided)
Pearson Chi-Square	1.696[a]	2	.428
Likelihood Ratio	1.691	2	.429
Linear-by-Linear Association	.324	1	.569
N of Valid Cases	421		

a. 0 cells (.0%) have expected count less than 5. The minimum expected count is 22.00.

GPA2 * SC6

Crosstab

			SC6		
			Yes	No	Total
GPA2	< 3.75	Count	40	8	48
		% within SC6	9.8%	66.7%	11.4%
	3.75 to 4.49	Count	186	4	190
		% within SC6	45.5%	33.3%	45.1%
	4.50 to 5.0	Count	183	0	183
		% within SC6	44.7%	.0%	43.5%
Total		Count	409	12	421
		% within SC6	100.0%	100.0%	100.0%

Chi-Square Tests

	Value	df	Asymp. Sig. (2-sided)
Pearson Chi-Square	38.839[a]	2	.000
Likelihood Ratio	26.985	2	.000
Linear-by-Linear Association	26.942	1	.000
N of Valid Cases	421		

a. 1 cells (16.7%) have expected count less than 5. The minimum expected count is 1.37.

GPA2 * SCC6

Crosstab

			SCC6		
			Yes	No	Total
GPA2	< 3.75	Count	5	43	48
		% within SCC6	25.0%	10.7%	11.4%
	3.75 to 4.49	Count	8	182	190
		% within SCC6	40.0%	45.4%	45.1%
	4.50 to 5.0	Count	7	176	183
		% within SCC6	35.0%	43.9%	43.5%
Total		Count	20	401	421
		% within SCC6	100.0%	100.0%	100.0%

Chi-Square Tests

	Value	df	Asymp. Sig. (2-sided)
Pearson Chi-Square	3.874[a]	2	.144
Likelihood Ratio	3.074	2	.215
Linear-by-Linear Association	2.288	1	.130
N of Valid Cases	421		

a. 1 cells (16.7%) have expected count less than 5. The minimum expected count is 2.28.

GPA2 * SC7

Crosstab

			SC7		Total
			Yes	No	
GPA2	< 3.75	Count	33	15	48
		% within SC7	11.0%	12.4%	11.4%
	3.75 to 4.49	Count	132	58	190
		% within SC7	44.0%	47.9%	45.1%
	4.50 to 5.0	Count	135	48	183
		% within SC7	45.0%	39.7%	43.5%
Total		Count	300	121	421
		% within SC7	100.0%	100.0%	100.0%

Chi-Square Tests

	Value	df	Asymp. Sig. (2-sided)
Pearson Chi-Square	1.007[a]	2	.604
Likelihood Ratio	1.011	2	.603
Linear-by-Linear Association	.873	1	.350
N of Valid Cases	421		

a. 0 cells (.0%) have expected count less than 5. The minimum expected count is 13.80.

GPA2 * SCC7

Crosstab

			SCC7		Total
			Yes	No	
GPA2	< 3.75	Count	20	28	48
		% within SCC7	12.3%	10.8%	11.4%
	3.75 to 4.49	Count	79	111	190
		% within SCC7	48.8%	42.9%	45.1%
	4.50 to 5.0	Count	63	120	183
		% within SCC7	38.9%	46.3%	43.5%
Total		Count	162	259	421
		% within SCC7	100.0%	100.0%	100.0%

Chi-Square Tests

	Value	df	Asymp. Sig. (2-sided)
Pearson Chi-Square	2.247[a]	2	.325
Likelihood Ratio	2.257	2	.324
Linear-by-Linear Association	1.797	1	.180
N of Valid Cases	421		

a. 0 cells (.0%) have expected count less than 5. The minimum expected count is 18.47.

GPA2 * SC8

Crosstab

			SC8		Total
			Yes	No	
GPA2	< 3.75	Count	29	19	48
		% within SC8	8.2%	28.4%	11.4%
	3.75 to 4.49	Count	154	36	190
		% within SC8	43.5%	53.7%	45.1%
	4.50 to 5.0	Count	171	12	183
		% within SC8	48.3%	17.9%	43.5%
Total		Count	354	67	421
		% within SC8	100.0%	100.0%	100.0%

Chi-Square Tests

	Value	df	Asymp. Sig. (2-sided)
Pearson Chi-Square	33.374[a]	2	.000
Likelihood Ratio	31.503	2	.000
Linear-by-Linear Association	32.224	1	.000
N of Valid Cases	421		

a. 0 cells (.0%) have expected count less than 5. The minimum expected count is 7.64.

GPA2 * SCC8

Crosstab

			SCC8		Total
			Yes	No	
GPA2	< 3.75	Count	14	34	48
		% within SCC8	23.0%	9.4%	11.4%
	3.75 to 4.49	Count	29	161	190
		% within SCC8	47.5%	44.7%	45.1%
	4.50 to 5.0	Count	18	165	183
		% within SCC8	29.5%	45.8%	43.5%
Total		Count	61	360	421
		% within SCC8	100.0%	100.0%	100.0%

Chi-Square Tests

	Value	df	Asymp. Sig. (2-sided)
Pearson Chi-Square	11.636[a]	2	.003
Likelihood Ratio	10.416	2	.005
Linear-by-Linear Association	10.386	1	.001
N of Valid Cases	421		

a. 0 cells (.0%) have expected count less than 5. The minimum expected count is 6.95.

GPA2 * SC9

Crosstab

			SC9		Total
			Yes	No	
GPA2	< 3.75	Count	42	6	48
		% within SC9	10.3%	42.9%	11.4%
	3.75 to 4.49	Count	185	5	190
		% within SC9	45.5%	35.7%	45.1%
	4.50 to 5.0	Count	180	3	183
		% within SC9	44.2%	21.4%	43.5%
Total		Count	407	14	421
		% within SC9	100.0%	100.0%	100.0%

Chi-Square Tests

	Value	df	Asymp. Sig. (2-sided)
Pearson Chi-Square	14.470[a]	2	.001
Likelihood Ratio	9.800	2	.007
Linear-by-Linear Association	9.273	1	.002
N of Valid Cases	421		

a. 1 cells (16.7%) have expected count less than 5. The minimum expected count is 1.60.

GPA2 * SCC9

Crosstab

			SCC9		Total
			Yes	No	
GPA2	< 3.75	Count	15	33	48
		% within SCC9	14.0%	10.5%	11.4%
	3.75 to 4.49	Count	51	139	190
		% within SCC9	47.7%	44.3%	45.1%
	4.50 to 5.0	Count	41	142	183
		% within SCC9	38.3%	45.2%	43.5%
Total		Count	107	314	421
		% within SCC9	100.0%	100.0%	100.0%

Chi-Square Tests

	Value	df	Asymp. Sig. (2-sided)
Pearson Chi-Square	1.941[a]	2	.379
Likelihood Ratio	1.925	2	.382
Linear-by-Linear Association	1.937	1	.164
N of Valid Cases	421		

a. 0 cells (.0%) have expected count less than 5. The minimum expected count is 12.20.

GPA2 * SC10

Crosstab

			SC10		Total
			Yes	No	
GPA2	< 3.75	Count	42	6	48
		% within SC10	10.4%	35.3%	11.4%
	3.75 to 4.49	Count	182	8	190
		% within SC10	45.0%	47.1%	45.1%
	4.50 to 5.0	Count	180	3	183
		% within SC10	44.6%	17.6%	43.5%
Total		Count	404	17	421
		% within SC10	100.0%	100.0%	100.0%

Chi-Square Tests

	Value	df	Asymp. Sig. (2-sided)
Pearson Chi-Square	11.602[a]	2	.003
Likelihood Ratio	9.299	2	.010
Linear-by-Linear Association	9.796	1	.002
N of Valid Cases	421		

a. 1 cells (16.7%) have expected count less than 5. The minimum expected count is 1.94.

GPA2 * SCC10

Crosstab

			SCC10		Total
			Yes	No	
GPA2	< 3.75	Count	14	34	48
		% within SCC10	16.3%	10.1%	11.4%
	3.75 to 4.49	Count	40	150	190
		% within SCC10	46.5%	44.8%	45.1%
	4.50 to 5.0	Count	32	151	183
		% within SCC10	37.2%	45.1%	43.5%
Total		Count	86	335	421
		% within SCC10	100.0%	100.0%	100.0%

Chi-Square Tests

	Value	df	Asymp. Sig. (2-sided)
Pearson Chi-Square	3.275[a]	2	.194
Likelihood Ratio	3.118	2	.210
Linear-by-Linear Association	2.999	1	.083
N of Valid Cases	421		

a. 0 cells (.0%) have expected count less than 5. The minimum expected count is 9.81.

GPA2 * SC11

Crosstab

			SC11		Total
			Yes	No	
GPA2	< 3.75	Count	45	3	48
		% within SC11	11.1%	21.4%	11.4%
	3.75 to 4.49	Count	183	7	190
		% within SC11	45.0%	50.0%	45.1%
	4.50 to 5.0	Count	179	4	183
		% within SC11	44.0%	28.6%	43.5%
Total		Count	407	14	421
		% within SC11	100.0%	100.0%	100.0%

Chi-Square Tests

	Value	df	Asymp. Sig. (2-sided)
Pearson Chi-Square	2.092[a]	2	.351
Likelihood Ratio	1.933	2	.380
Linear-by-Linear Association	2.013	1	.156
N of Valid Cases	421		

a. 1 cells (16.7%) have expected count less than 5. The minimum expected count is 1.60.

GPA2 * SCC11

Crosstab

			SCC11		Total
			Yes	No	
GPA2	< 3.75	Count	10	38	48
		% within SCC11	13.7%	10.9%	11.4%
	3.75 to 4.49	Count	32	158	190
		% within SCC11	43.8%	45.4%	45.1%
	4.50 to 5.0	Count	31	152	183
		% within SCC11	42.5%	43.7%	43.5%
Total		Count	73	348	421
		% within SCC11	100.0%	100.0%	100.0%

Chi-Square Tests

	Value	df	Asymp. Sig. (2-sided)
Pearson Chi-Square	.462[a]	2	.794
Likelihood Ratio	.443	2	.801
Linear-by-Linear Association	.215	1	.643
N of Valid Cases	421		

a. 0 cells (.0%) have expected count less than 5. The minimum expected count is 8.32.

GPA2 * SC12

Crosstab

			SC12		Total
			Yes	No	
GPA2	< 3.75	Count	45	3	48
		% within SC12	11.1%	21.4%	11.4%
	3.75 to 4.49	Count	183	7	190
		% within SC12	45.0%	50.0%	45.1%
	4.50 to 5.0	Count	179	4	183
		% within SC12	44.0%	28.6%	43.5%
Total		Count	407	14	421
		% within SC12	100.0%	100.0%	100.0%

Chi-Square Tests

	Value	df	Asymp. Sig. (2-sided)
Pearson Chi-Square	2.092[a]	2	.351
Likelihood Ratio	1.933	2	.380
Linear-by-Linear Association	2.013	1	.156
N of Valid Cases	421		

a. 1 cells (16.7%) have expected count less than 5. The minimum expected count is 1.60.

GPA2 * SCC12

Crosstab

			SCC12		Total
			Yes	No	
GPA2	< 3.75	Count	14	34	48
		% within SCC12	12.1%	11.1%	11.4%
	3.75 to 4.49	Count	45	145	190
		% within SCC12	38.8%	47.5%	45.1%
	4.50 to 5.0	Count	57	126	183
		% within SCC12	49.1%	41.3%	43.5%
Total		Count	116	305	421
		% within SCC12	100.0%	100.0%	100.0%

Chi-Square Tests

	Value	df	Asymp. Sig. (2-sided)
Pearson Chi-Square	2.672[a]	2	.263
Likelihood Ratio	2.687	2	.261
Linear-by-Linear Association	.897	1	.344
N of Valid Cases	421		

a. 0 cells (.0%) have expected count less than 5. The minimum expected count is 13.23.

GPA2 * SC13

Crosstab

			SC13		Total
			Yes	No	
GPA2	< 3.75	Count	44	4	48
		% within SC13	10.7%	40.0%	11.4%
	3.75 to 4.49	Count	185	5	190
		% within SC13	45.0%	50.0%	45.1%
	4.50 to 5.0	Count	182	1	183
		% within SC13	44.3%	10.0%	43.5%
Total		Count	411	10	421
		% within SC13	100.0%	100.0%	100.0%

Chi-Square Tests

	Value	df	Asymp. Sig. (2-sided)
Pearson Chi-Square	10.041[a]	2	.007
Likelihood Ratio	8.369	2	.015
Linear-by-Linear Association	8.829	1	.003
N of Valid Cases	421		

a. 3 cells (50.0%) have expected count less than 5. The minimum expected count is 1.14.

GPA2 * SCC14

Crosstab

			SCC14		Total
			Yes	No	
GPA2	< 3.75	Count	5	43	48
		% within SCC14	12.8%	11.3%	11.4%
	3.75 to 4.49	Count	23	167	190
		% within SCC14	59.0%	43.7%	45.1%
	4.50 to 5.0	Count	11	172	183
		% within SCC14	28.2%	45.0%	43.5%
Total		Count	39	382	421
		% within SCC14	100.0%	100.0%	100.0%

Chi-Square Tests

	Value	df	Asymp. Sig. (2-sided)
Pearson Chi-Square	4.205[a]	2	.122
Likelihood Ratio	4.353	2	.113
Linear-by-Linear Association	2.676	1	.102
N of Valid Cases	421		

a. 1 cells (16.7%) have expected count less than 5. The minimum expected count is 4.45.

GPA2 * SCC13

Crosstab

			SCC13		Total
			Yes	No	
GPA2	< 3.75	Count	16	32	48
		% within SCC13	23.2%	9.1%	11.4%
	3.75 to 4.49	Count	30	160	190
		% within SCC13	43.5%	45.5%	45.1%
	4.50 to 5.0	Count	23	160	183
		% within SCC13	33.3%	45.5%	43.5%
Total		Count	69	352	421
		% within SCC13	100.0%	100.0%	100.0%

Chi-Square Tests

	Value	df	Asymp. Sig. (2-sided)
Pearson Chi-Square	12.056[a]	2	.002
Likelihood Ratio	10.363	2	.006
Linear-by-Linear Association	8.873	1	.003
N of Valid Cases	421		

a. 0 cells (.0%) have expected count less than 5. The minimum expected count is 7.87.

GPA2 * SC14

Crosstab

			SC14		Total
			Yes	No	
GPA2	< 3.75	Count	27	21	48
		% within SC14	12.4%	10.3%	11.4%
	3.75 to 4.49	Count	95	95	190
		% within SC14	43.8%	46.6%	45.1%
	4.50 to 5.0	Count	95	88	183
		% within SC14	43.8%	43.1%	43.5%
Total		Count	217	204	421
		% within SC14	100.0%	100.0%	100.0%

Chi-Square Tests

	Value	df	Asymp. Sig. (2-sided)
Pearson Chi-Square	.617[a]	2	.735
Likelihood Ratio	.618	2	.734
Linear-by-Linear Association	.053	1	.817
N of Valid Cases	421		

a. 0 cells (.0%) have expected count less than 5. The minimum expected count is 23.26.
